# Comparative transcriptome analysis revealed molecular mechanisms of peanut leaves responding to *Ralstonia solanacearum* and its type III secretion system mutant

**DOI:** 10.3389/fmicb.2022.998817

**Published:** 2022-08-25

**Authors:** Yong Yang, Ting Chen, Xiaoqiu Dai, Dong Yang, Yushuang Wu, Huilan Chen, Yixiong Zheng, Qingqing Zhi, Xiaorong Wan, Xiaodan Tan

**Affiliations:** ^1^Guangzhou Key Laboratory for Research and Development of Crop Germplasm Resources, Zhongkai University of Agriculture and Engineering, Guangzhou, China; ^2^Key Laboratory of Horticultural Plant Biology (HZAU), Ministry of Education, Key Laboratory of Potato Biology and Biotechnology (HZAU), Ministry of Agriculture and Rural Affairs, Huazhong Agricultural University, Wuhan, China

**Keywords:** peanut, *Ralstonia solanacearum*, DEGs, RNA sequencing, PAMPs, T3Es

## Abstract

Bacterial wilt caused by *Ralstonia solanacearum* is a serious soil-borne disease that limits peanut production and quality, but the molecular mechanisms of the peanut response to *R. solanacearum* remain unclear. In this study, we reported the first work analyzing the transcriptomic changes of the resistant and susceptible peanut leaves infected with *R. solanacearum* HA4-1 and its type III secretion system mutant strains by the cutting leaf method at different timepoints (0, 24, 36, and 72 h post inoculation). A total of 125,978 differentially expressed genes (DEGs) were identified and subsequently classified into six groups to analyze, including resistance-response genes, susceptibility-response genes, PAMPs induced resistance-response genes, PAMPs induced susceptibility-response genes, T3Es induced resistance-response genes, and T3Es induced susceptibility-response genes. KEGG enrichment analyses of these DEGs showed that plant-pathogen interaction, plant hormone signal transduction, and MAPK signaling pathway were the outstanding pathways. Further analysis revealed that CMLs/CDPKs-WRKY module, MEKK1-MKK2-MPK3 cascade, and auxin signaling played important roles in the peanut response to *R. solanacearum*. Upon *R. solanacearum infection* (RSI), three early molecular events were possibly induced in peanuts, including Ca^2+^ activating CMLs/CDPKs-WRKY module to regulate the expression of resistance/susceptibility-related genes, auxin signaling was induced by AUX/IAA-ARF module to activate auxin-responsive genes that contribute to susceptibility, and MEKK1-MKK2-MPK3-WRKYs was activated by phosphorylation to induce the expression of resistance/susceptibility-related genes. Our research provides new ideas and abundant data resources to elucidate the molecular mechanism of the peanut response to *R. solanacearum* and to further improve the bacterial wilt resistance of peanuts.

## Introduction

Peanut (*Arachis hypogaea* L) is an important economic and oil crop in the world (Pandey et al., 2012; Varshney et al., 2013). However, the yield and quality of peanut are severely devastated by bacterial wilt (BW). BW, caused by *Ralstonia solanacearum*, is a very destructive soil-borne bacterial disease. *Ralstonia solanacearum*, with a wide host range, can infect more than 200 plant species in 54 families, such as peanut, potato (*Solanum tuberosum*), ginger (*Zingiber officinale*), and patchouli (*Pogostemon cablin*; Jiang et al., 2017; Sun et al., 2022). The economic losses caused by BW are difficult to estimate every year in the world (Genin, 2010; Deberdt et al., 2021). BW has happened in most of the 13 main peanut-producing provinces in China, which could cause up to 50%–100% of yield losses (Jiang et al., 2017). To date, the breeding of resistant peanut cultivars is the most efficient strategy to control this disease, which is inexpensive and environmentally friendly (Liao et al., 2005; Huet, 2014). Discovery of resistant genes is the foundation for breeding resistant cultivars, which also provides theoretical basis to understand in detail the mechanisms of the peanut response to *R. solanacearum*. However, resistant genes and molecular mechanisms of the peanut response to *R. solanacearum* remain largely unknown.

During plant-pathogen interactions, plants have evolved a two-layer innate immune system to defend against pathogens’ attack (Jones and Dangl, 2006; Thomma et al., 2011). When pathogens contact with plants, the pattern-recognition receptors (PRRs) on the plant cell surface recognize the pathogen-associated molecular patterns (PAMPs), which triggers the PAMP-triggered immunity (PTI) to prevent the invasion of pathogens. Some pathogens can inhibit PTI by injecting type III effectors (T3Es) into host cells to trigger more powerful attacks. In resistant plants, some T3Es are recognized by resistance proteins to activate effector-triggered immunity (ETI), which is another important resistant response. The T3E being recognized is called the avirulence effector, and the gene responsible for recognizing the avirulence effector is called the resistance gene. With the PTI and ETI initiated, a set of resistant responses on transcriptome level will be activated to restrain the propagation of the pathogens (Dong, 1998; Bonas and Lahaye, 2002; Wise et al., 2007; Göhre and Robatzek, 2008). In susceptible plants, T3Es, called virulence effectors, are injected into host cells by pathogens to induce the susceptibility response. A series of susceptibility-response genes will be induced to help the invasion of the pathogens in the process. Recently, T3Es-related plant resistance and susceptibility proteins were identified by proteomic analysis of potato responding to the invasion of *R. solanacearum* UW551 and its type III secretion system (T3SS) mutant (Wang et al., 2021). *Ralstonia solanacearum* T3Es are secreted by the T3SS, which is like a pinhead device. When *R. solanacearum* interacts with plants, most of the T3Es can be directly injected into plant cells through the T3SS (Tampakaki et al., 2010; Coll and Valls, 2013). In *R. solanacearum*, most of the T3Es genes have a TTCGn16TTCG box (hrpII box) in the upstream sequence, which can be regulated and recognized by the HrpB protein. If HrpB is mutated, then the pathogen will be unable to secrete T3Es, resulting in a loss of pathogenicity (Boucher et al., 1985; Cunnac et al., 2004). Studies on the genes involved in PTI and ETI are not only helpful to discover the resistance and susceptibility (or related) genes, but also beneficial to reveal the mechanism of the plant response to pathogen.

Although more than 150 disease resistance genes have been identified in plants, knowledge about the genes resistant to BW is still limited (Osuna-Cruz et al., 2018). To date, resistance proteins RRS1-R/RPS4 and ERECTA, involved in resistance to BW, have been identified in *Arabidopsis thaliana* (Deslandes et al., 2002; Godiard et al., 2003; Saucet et al., 2015). There were no studies on the peanut BW resistance genes reported thus far. Current research mainly focused on resistance marker screening for BW and transcriptome changes after *R. solanacearum* inoculation to discover possible resistance genes. Some major quantitative trait locus (QTLs) for BW resistance were identified by different approaches in peanut (Zhao et al., 2016; Luo et al., 2019). In order to reveal the molecular basis of peanut resistance to *R. solanacearum*, microarray analysis and RNA sequencing (RNA-Seq) were employed to identify DEGs of the roots with *R. solanacearum* infection (RSI) in *A. duranensis* and *A. hypogaea*, respectively (Chen et al., 2013a, 2014). KEGG analysis revealed that these DEGs were mainly involved in the biosynthetic pathways of terpenoids and flavonoids in *A. duranensis* and the primary metabolisms in *A. hypogaea*. There was only the above one report about the transcriptome changes analysis of peanut roots with RSI at present, and transcriptome analysis of peanut leaves with RSI was lacking. Peanut BW resistance-related genes *AhRLK1*, *AhRRS5*, and *AhGLK1b* were identified through microarray analysis (Zhang et al., 2017, 2019; Ali et al., 2020). The overexpression of the three genes in tobacco enhanced BW resistance of the transgenic lines. Therefore, more efforts are needed to reveal the peanut BW resistance and susceptibility (or related) genes, which will help to uncover the molecular mechanism of the peanut response to *R. solanacearum* and provide the best strategy for breeding BW-resistant peanut cultivars.

Cultivated peanut (AABB, 2*n* = 40) is allotetraploid and formed by hybridization between diploid *Arachis duranensis* (AA, 2*n* = 20) and *Arachis ipaensis* (BB, 2*n* = 20; Zhuang et al., 2019). Due to the complexity of the cultivated peanut genome, it is difficult to isolate BW resistance genes by map-based cloning. RNA sequencing technology is a more comprehensive and efficient method for exploring the mechanism of the peanut response to *R. solanacearum*, which had been successfully performed to research seed development and stress response in peanuts (Guimarães et al., 2012; Zhang et al., 2012; Chen et al., 2013b). Comparative transcriptome analysis using RNA sequencing (RNA-Seq) has been widely used to investigate the transcriptional changes of different tissues infected by *R. solanacearum* in other plants, such as *Arabidopsis* roots and aerial parts (Hu et al., 2008; Feng et al., 2012; Zhao et al., 2019), tomato roots and stems (Ishihara et al., 2012; French et al., 2018), the wild potato roots (Zuluaga et al., 2015), and tobacco stems (Pan et al., 2021). The aforementioned studies demonstrated that the transcriptional reprogramming was different between aerial parts and roots. Bacteria need to break through many barriers before entering the vascular system from outside the root, while by inoculating leaves, they can enter the vascular system and interact with plant cells directly. Leaf inoculation, which bypass the root, can avoid infection randomness and is conducive to accurate study (Xue et al., 2020). In peanut and tobacco, some important genes response to BW were identified by the expression analysis of leaves with RSI, and the functions of these genes were verified later (Kiba et al., 2007; Maimbo et al., 2010; Makoto et al., 2015; Zhang et al., 2017, 2019; Ali et al., 2020). RNA-seq analysis of peanut roots with RSI had been performed in a previous study (Chen et al., 2014). However, available information concerning RNA-seq analysis of peanut leaves with RSI is limited.

In this study, we identified the resistant and susceptible cultivated peanut A165 and A281 from 110 peanut germplasm resources. The physiological and transcriptional changes were investigated in the A165 and A281 leaves with RSI. Bioinformatics analysis was performed to identify a large number of resistance- and susceptibility-related genes in peanut leaves upon RSI. This study will provide valuable information for elucidating the complex regulatory networks associated with the peanut response to *R. solanacearum*.

## Materials and methods

### Plant materials and bacterial strains

Two peanut cultivars, resistant cultivar A165 and susceptible cultivar A281, which belong to cultivars of southern China, are preserved in Zhongkai University of Agriculture and Engineering. Peanut seeds were germinated and grown as described by our previous study (Tan et al., 2022). Healthy peanut plants with seven-to-eight full grown leaves were used for *R. solanacearum* inoculation experiments.

The *R. solanacearum* strain HA4-1 isolated from peanut plants, which belongs to phylotype I sequevar 14 M biovar 3, was used for inoculation (Tan et al., 2019). The *ΔhrpB* mutant of HA4-1 was generated according to recent study (Wang et al., 2021). The *hrpB* gene was replaced with a cassette harboring spectinomycin (*Spe*) resistance. The genome sequence containing the *hrpB* gene and its two flanking regions (500–1,000 bp) was amplified from HA4-1 genome and then inserted into pEASY-Blunt vector. A reverse amplification of the vector was performed to remove the *hrpB* gene that generates a linear vector with the flanking regions of the *hrpB* gene. The *Spe* gene was connected into the above linear vector to replace the *hrpB* gene. Then the *Spe* cassette with the *hrpB* flanking regions was generated, which was used to be transformed into *R. solanacearum* competent cells to remove the *hrpB* gene. Bacterial suspension for inoculation experiments was prepared, as described by the previous studies (Boucher et al., 1985; Tan et al., 2022).

### Inoculation and sampling

The cutting leaf method (Supplementary Figure S1), which was performed as reported (Tan et al., 2022), was used for peanut plant inoculation. More than 45 plants from each peanut line were inoculated with HA4-1. The leaves of the control plants were inoculated with *ΔhrpB* mutant, and sterile water. Three biological replicates were set with more than 15 plants per replicate. The disease score of each plant was recorded every day consecutively for 20 days after inoculation. Statistical analysis of disease index and survival ratio was operated as described in the previous studies (Remigi et al., 2011; Tan et al., 2022).

More than 48 plants from each peanut line were inoculated for transcriptomic analysis. The two-third of the leaflets, which were inoculated with HA4-1 and *ΔhrpB* mutant, were collected at 0, 24, 36, and 72 hpi, respectively. Three randomly selected peanut plants were sampled at every time point. All the four leaflets of each plant were used as a biological replicate. A total of 48 samples were collected for further analysis.

### Bacterial population detection

Two-thirds of the leaflet from the inverted third leaf was selected to detect bacterial population at 0, 24, 36, 48, 72, and 96 hpi with *R. solanacearum* (HA4-1 and *ΔhrpB* mutant) for each peanut line. The leaflets were soaked with 75% ethanol absolute for 4 min to disinfect the surface, and washed in sterile water for 10 min. One square centimeter of the clean leaflet was vigorously ground to powder in a mortar, and soaked in 10 ml sterile water for 30 min. Serial dilutions of the suspensions were made for spread plate count. 100 μl aliquots were spread on the surface of CPG agar medium containing TTC and incubated at 28°C for 48 h. The ingredient of medium was the same as previously reported (Tan et al., 2022). The number of *R. solanacearum* colonies was counted to calculate cfu per cm^2^ of the leaflet. Six randomly selected peanut plants as different replicates were sampled for every time point.

### Electrolyte leakage measurement

Two-thirds of leaflet from the inverted third leaf was selected to measure electrolyte leakage at 0, 24, 36, 48, 72, and 96 hpi with *R. solanacearum* (HA4-1 and *ΔhrpB* mutant) for each peanut line. Six pieces per leaflet were excised with a puncher of 6 mm diameter. They were washed thoroughly with sterile water, and soaked in 20 ml sterile water for 3 h. The electrical conductivity of the suspensions (EL1) was measured using a conductivity meter (DDS-11A, Leica Instrument Factory, Shanghai, China). The above suspension was boiled at 90°C for 30 min, cooled at room temperature, and the electrical conductivity (EL2) was measured again. The electrolyte leakage was calculated by this equation: EL (%) = (EL1/EL2) × 100 (Alexander et al., 2020). Six randomly selected peanut plants as different replicates were sampled for every time point.

### RNA sequencing

All samples were sent to Shanghai Personal Biotechnology Cp. Ltd. for RNA sequencing. Total RNA was extracted using the Trizol Reagent (Invitrogen Life Technologies) according to the manufacturer’s instructions. The quality and integrity of RNA were determined using a NanoDrop spectrophotometer (Thermo Scientific). RNA sequencing was performed on NovaSeq 6000 platform (Illumina).

A total of 3 μg of RNA was prepared from each sample for cDNA library construction. Beads with oligo (dT) were used to purify mRNA. mRNA was interrupted to short fragments by using divalent cations under elevated temperature in an Illumina proprietary fragmentation buffer. The first strand cDNA was synthesized using random oligonucleotides and Super Script II. Second strand cDNA synthesis was subsequently performed using buffer, dNTPs, RNaseH, and DNA polymerase I. Remaining overhangs were converted into blunt ends using exonuclease/polymerase. After the 3′ ends were adenylated, Illumina PE adapter oligonucleotides were ligated to the DNA fragments for hybridization. The cDNA fragments of the preferred 400–500 bp in length were selected and purified using the AMPure XP system (Beckman Coulter, Beverly, CA, United States). DNA fragments with ligated adaptor molecules on both ends were selectively enriched using Illumina PCR Primer Cocktail in a 15 cycle PCR reaction. The library products were purified using AMPure XP system and quantified using the Agilent high sensitivity DNA assay on a Bioanalyzer 2,100 system (Agilent).

### Bioinformatics analysis

The RNA-Seq raw sequencing data were filtered using the Cutadapt (v1.15) software.^1^ The filtered clean reads were further mapped to the peanut reference genome^2^ using HISAT2v2.0.5^3^ with up to two mismatches allowed. The difference expression of genes was analyzed by DESeq (1.30.0)^4^ with a screened condition of |log2 fold-change| > 1.0 and significant *p*-value < 0.05. All DEGs were mapped to the Gene Ontology (GO) database and the Kyoto Encyclopedia of Genes and Genomes (KEGG) database to identify the main GO Terms and metabolic pathways, respectively. Other bioinformatics analysis was performed on the website.^5^

### qRT-PCR analysis

Samples for BW resistance-related-gene expression analysis were collected at 0, 6, 12, 24, 36, 48, 72, and 96 hpi with *R. solanacearum* HA4-1 for each peanut line. Total RNA was extracted through Plant RNA Mini Kit (Magen Biotechnology, Guangzhou, China) following the manufacturer’s protocol. Reverse transcription was performed with EasyScript^®^ One-Step gDNA Removal and cDNA Synthesis SuperMix (TransGen Biotech, Beijing, China) in accordance with the manufacturer’s instructions. RT-PCR was performed on the CFX96TM Real-Time PCR System (Link-Bio, Beijing, China) by using PerfectStart™ Green qPCR SuperMix (Magen Biotechnology, Guangzhou, China). The parameters of thermal cycle were 94°C for 30 s, followed by 40 cycles of 94°C for 5 s, 60°C for 30 s at a volume of 10 μl. The relative gene expression level was calculated using the 2^−ΔΔCT^ method with normalization to the internal reference peanut actin gene. As the same with transcriptomic analysis, three biological replicates were set with one plant per replicate. All reactions for each gene were performed in triplicate. Gene-specific primers used for qPCR were designed with Primer Premier 5 software (Palo Alto, CA, United States) according to *A. hypogaea* cDNA sequences (Supplementary Table S1).

### Statistical analysis

Statistical analyses and graphs were generated by using the GraphPad Prism 8.0 software.

## Results

### Characterization of peanut phenotype following RSI

**Figure 1:**
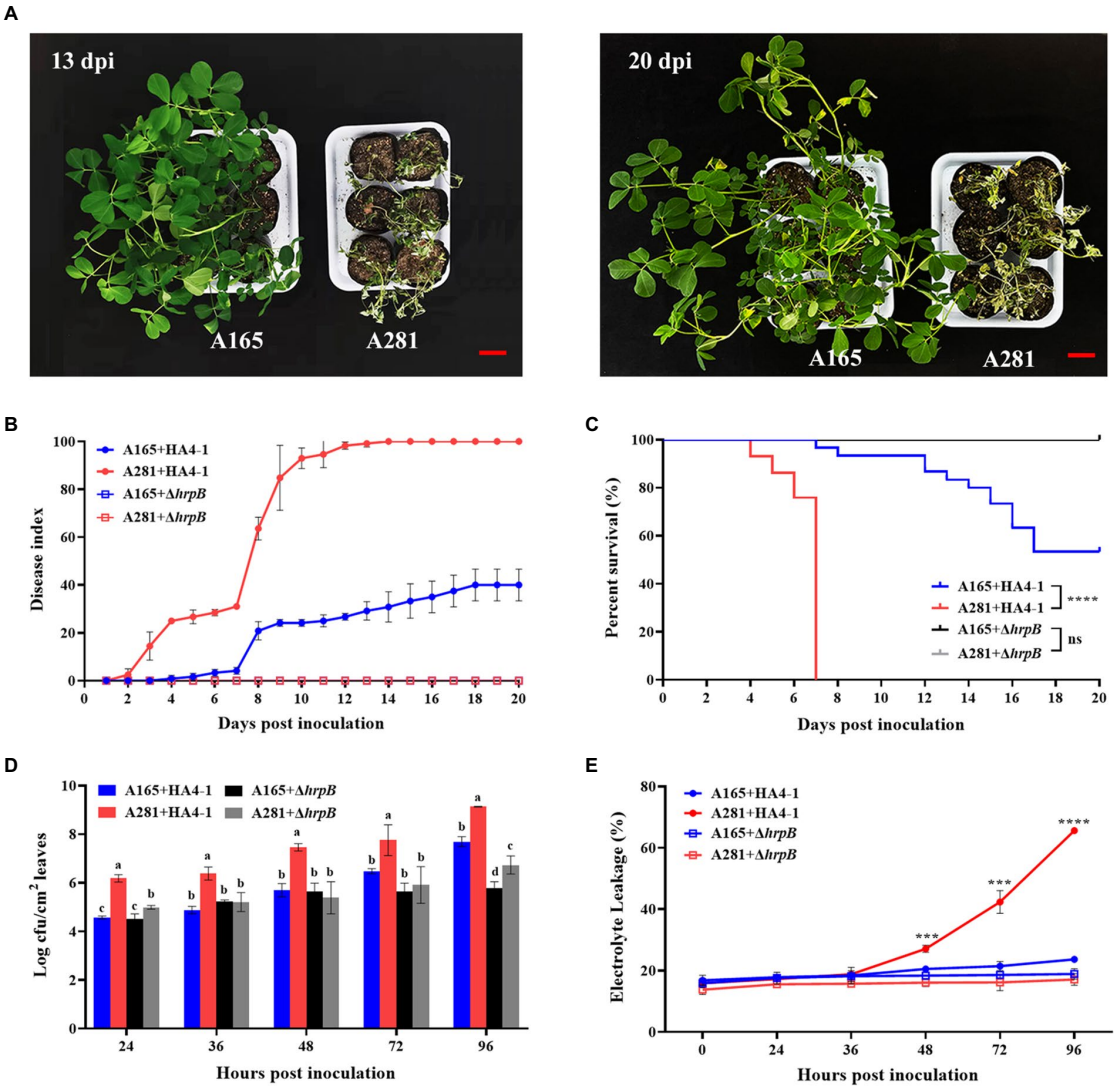
The phenotype of A165 and A281 inoculated with *R. solanacearum* HA4-1 by the cutting leaf method. **(A)** Disease symptoms of A165 and A281 inoculated with HA4-1 at 13 dpi and 20 dpi. Bar = 5 cm. **(B)** Disease index of A165 and A281 inoculated with HA4-1 and *ΔhrpB* mutant (mean ± SE). **(C)** Survival analysis of A165 and A281 inoculated with HA4-1 and *ΔhrpB* mutant (^****^p < 0.0001, “ns” means no significant difference). **(D)** *R. solanacearum* populations in leaf tissues of A165 and A281 inoculated with HA4-1 and *ΔhrpB* mutant. Different lowercases indicate significant differences according to ANOVA (mean ± SE, *p* < 0.05). **(E)** Electrolyte leakage in leaf tissues of A165 and A281 inoculated with HA4-1 and *ΔhrpB* mutant. The asterisks indicate significant differences between A165 and A281 according to Student’s *t*-test (mean ± SE, ^***^p < 0.001, ^****^p < 0.0001).

In a previous study, we successfully identified BW-resistant and-susceptible cultivated peanuts A165 and A281 by inoculating with *R. solanacearum* HA4-1 and PeaFJ1 according to the wilting symptoms (Tan et al., 2022). To confirm their phenotype, A165 and A281 were inoculated with HA4-1 and its *ΔhrpB* mutant by the cutting leaf method. A281 exhibited wilting symptoms on the inoculated leaves at 1 day post-inoculation (dpi) with HA4-1, while A165 displayed similar symptoms until 3 dpi (Supplementary Figure S2). Compared with A281 plants, A165 displayed attenuated disease symptoms (Figure 1A). At 13 dpi, all A281 plants inoculated with HA4-1 died (Figures 1A,B), while no A165 plant was wilted to death until 20 dpi (Figure 1A). Peanut plants inoculated with *ΔhrpB* mutant and sterile water displayed no disease symptoms at 13 dpi (Supplementary Figure S2). In order to ensure the reliability of the cutting leaf method, we also performed soil-drenching inoculation of A165 and A281 with HA4-1, and investigated the development of wilting symptoms associated with disease. Compared with the cutting leaf method, peanuts inoculated with the soil-drenching method showed similar disease phenotypes (Supplementary Figure S3), demonstrating that the cutting leaf method is convenient and effective for peanut BW study.

To further confirm disease response, we observed the differences of bacterial concentration and electrolyte leakage in the leaves of A165 and A281 inoculated by HA4-1 and *ΔhrpB* mutant (Figure 1D). The *R. solanacearum* concentration in the leaves of A165 and A281 was observed at 0, 24, 36, 48, 72, and 96 h post-inoculation (hpi) with HA4-1 and *ΔhrpB* mutant through plate counting. As expected, the *R. solanacearum* population was always significantly higher in the leaves of A281 inoculated by HA4-1 than in all other treatments at every timepoint. It suggested that the wild-type HA4-1 strains grew most rapidly in A281. When *ΔhrpB* mutant infected the peanut leaves, the bacterial concentration was significantly lower in A165 than in A281 at 24 and 96 hpi. There was no significant difference in bacterial concentration between the HA4-1 and the *ΔhrpB* mutant inoculated leaves of A165 at all timepoints except 96 hpi. It suggested that HA4-1 and *ΔhrpB* mutant have the same growth rate in A165 leaves at the early stages of infection. The electrolyte leakage in the leaves of A165 and A281 was measured at 0, 24, 36, 48, 72, and 96 hpi with HA4-1 and *ΔhrpB* mutant (Figure 1E). Before 48 hpi, there was no significant difference among all inoculation treatments. After that, the electrolyte leakage was always significantly higher in the leaves of A281 inoculated by HA4-1 than that in all other treatments. Meanwhile, the electrolyte leakage of the other three inoculation treatments did not fluctuate greatly from 0 to 96 hpi. It suggested that more cells died in A281 leaves infected by HA4-1. All the above results demonstrated that A165 was a clear BW resistance cultivar compared with A281 and *ΔhrpB* mutant was almost avirulent compared with the wild-type HA4-1.

### Determination of sampling timepoints for transcriptome analysis upon RSI

**Figure 2:**
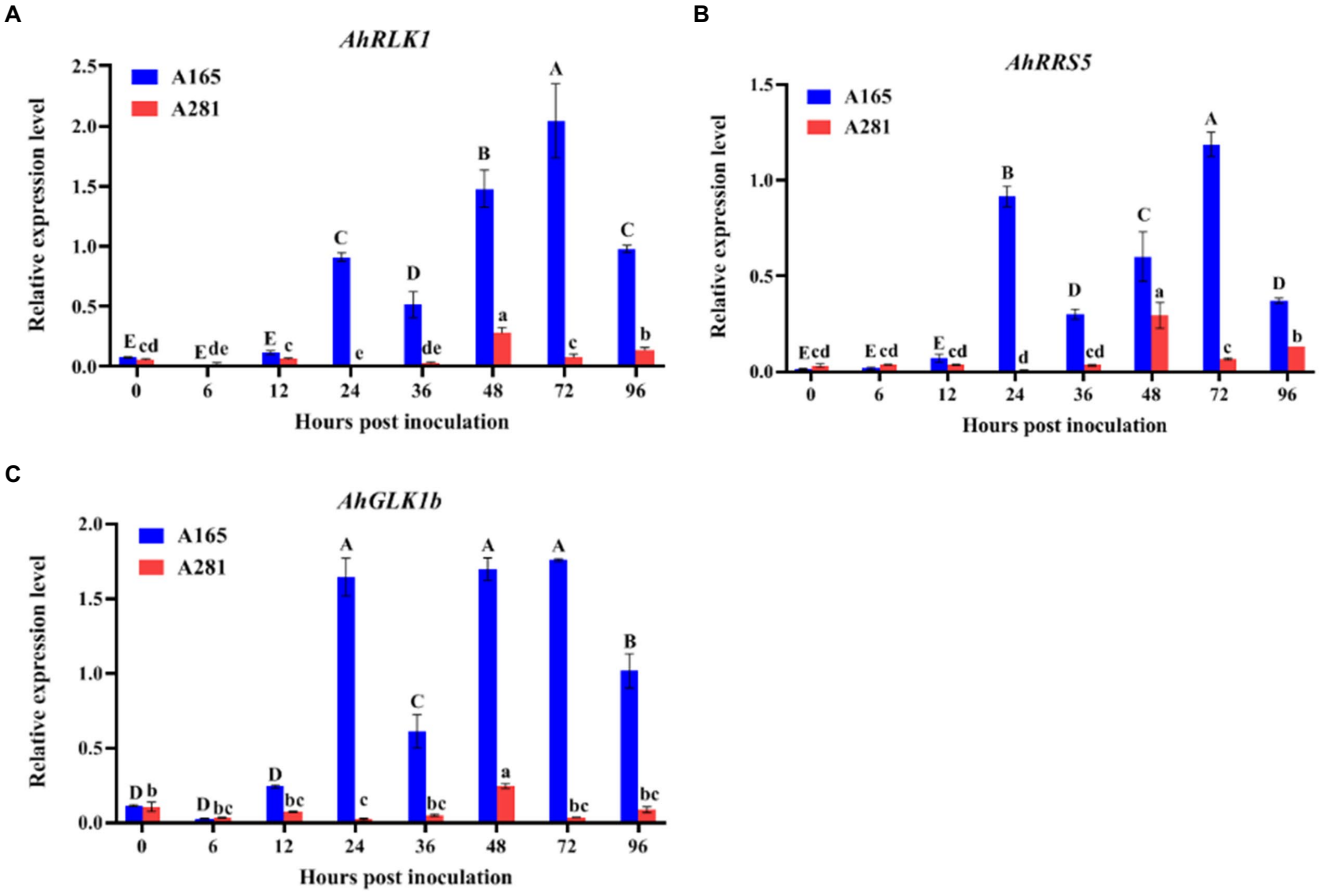
Expression analysis of *AhRLK1*, *AhRRS5* and *AhGLKb* in the leaves of A165 and A281 at 0, 6, 12, 24, 36, 48, 72, and 96 hpi with *R. solanacearum* HA4-1. Error bars represent standard errors. Different uppercases and lowercases indicate significant differences in the leaves of A165 and A281 at different timepoints according to ANOVA (*p* < 0.05), respectively.

Previously, by microarray analysis, three BW resistance-related genes *AhRLK1*, *AhRRS5,* and *AhGLKb* were identified in peanut (Zhang et al., 2017, 2019; Ali et al., 2020). To determine the sampling timepoints which are very important for transcriptome analysis, we analyzed the expression of these three genes in the leaves of A165 and A281 at 0, 6, 12, 24, 36, 48, 72, and 96 hpi with HA4-1 through quantitative real-time PCR (qRT-PCR). Their expression was significantly up-regulated in A165 leaves injected by HA4-1, and the expression trends were consistent over time, which display two peaks at 24 and 72 hpi (Figure 2). Meanwhile, the expression of the three genes was greatly lower in A281 than in A165, and there was no obvious expression trend in A281 at different timepoints. According to phenotype and qRT-PCR results, we thus selected 24, 36, and 72 hpi as the sampling timepoints for transcriptome analysis.

### Time series of global transcriptional reprogramming in peanut leaves challenged with *Ralstonia Solanacearum*

To understand the molecular mechanisms underlying the peanut response to *R. solanacearum*, the global transcripts of 14-day-old peanut seedling leaves infected by wild-type HA4-1 and its *hrpB* mutant were sequenced by the Illumina platform. Resistant and susceptible peanut leaves inoculated by HA4-1 and *ΔhrpB* mutant at multiple time points (0, 24, 36, and 72 h) were collected (leaves inoculated by HA4-1 were termed as R0, R24, R36, and R72 for resistant peanut and S0, S24, S36, and S72 for susceptible peanut, leaves inoculated by *ΔhrpB* mutant were termed as RH0, RH24, RH36, and RH72 for resistant peanut and SH0, SH24, SH36, and SH72 for susceptible peanut). Three independent biological replicates were sequenced and analyzed at every timepoint (48 samples in total). An average of 41.7 million clean reads (ranging from 35.9 to 48.0 million) with Q30 > 93.4% were obtained by data filtering in every sample (Supplementary Table S2). To reveal the differentially expressed genes (DEGs) during peanut-*R. solanacearum* interaction, we compared *R. solanacearum*-infected leaves transcriptomes at 24, 36, and 72 h with those at 0 h. In addition, transcriptomes of HA4-1-infected leaves transcriptomes at 24, 36, and 72 h were compared with those of *ΔhrpB*-treated leaves at corresponding timepoints, respectively. Eighteen comparison groups were thus produced. Based on the DEGs screen criteria (|log_2_fold-change| > 1, *p*-value < 0.05), we identified a total of 125,978 peanut genes as DEGs, among which 72,487 genes were up-regulated and 53,500 genes were down-regulated (Table 1). The detail information of DEGs is shown in Table 1 and Supplementary Table S3.

**Table 1:** The number of DEGs in different comparison groups.

**Comparison group**	**Up-regulated**	**Down-regulated**	**Total**
R24 vs. R0	3,943	1,002	4,945
R36 vs. R0	6,482	4,720	11,202
R72 vs. R0	3,779	1,068	4,847
S24 vs. S0	2,259	1,394	3,653
S36 vs. S0	7,194	7,463	14,657
S72 vs. S0	7,972	7,341	15,313
RH24 vs. RH0	3,350	572	3,922
RH36 vs. RH0	6,439	5,121	11,560
RH72 vs. RH0	2,090	1,374	3,464
SH24 vs. SH0	3,188	2,343	5,531
SH36 vs. SH0	5,333	5,128	10,461
SH72 vs. SH0	1,873	1,336	3,209
R24 vs. RH24	1,338	1,751	3,089
R36 vs. RH36	1,075	1,975	3,050
R72 vs. RH72	2,213	1,209	3,422
S24 vs. SH24	5,380	4,152	9,532
S36 vs. SH36	3,009	1,513	4,522
S72 vs. SH72	5,561	4,038	9,599
Total	72,478	53,500	125,978

R0, R24, R36, and R72 represent the resistant peanut leaves inoculated by HA4-1 at 0, 24, 36, and 72 h; S0, S24, S36, and S72 indicate the susceptible peanut leaves with HA4-1 inoculation at 0, 24, 36, and 72 h; RH0, RH24, RH36, and RH72 mean the resistant peanut leaves with *ΔhrpB* inoculation at 0, 24, 36, and 72 h; SH0, SH24, SH36, and SH72 represent the susceptible peanut leaves with *ΔhrpB* inoculation at 0, 24, 36, and 72 h.

### DEGs in the resistant and susceptible peanut leaves response to HA4-1 strain

In order to explore the key determinant genes of peanuts associated with different responses to wild-type *R. solanacearum*, we analyzed the up-regulated and down-regulated genes, respectively. Firstly, we analyzed the up-regulated genes in resistant peanuts and the down-regulated genes in susceptible peanuts. We collectively called these genes as resistance-response DEGs. After that, to screen the susceptibility-response DEGs, we analyzed the down-regulated genes in resistant peanuts and the up-regulated genes in susceptible peanuts.

#### Resistance-response DEGs

**Figure 3:**
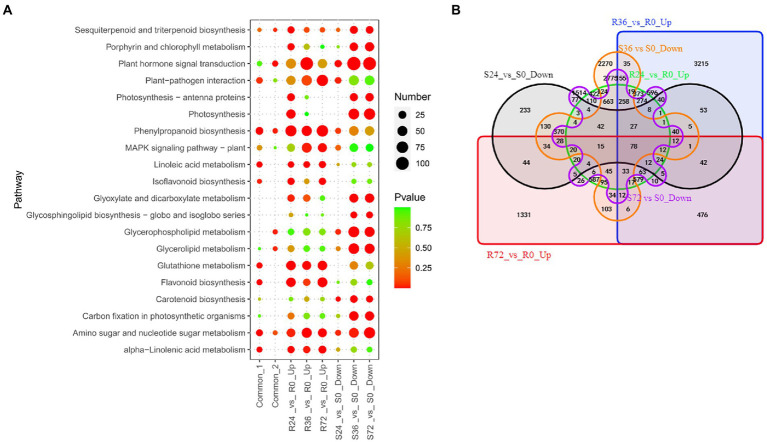
Identification of resistance-response DEGs in R24 vs. R0, R36 vs. R0, R72 vs. R0, S24 vs. S0, S36 vs. S0, and S72 vs. S0. **(A)** KEGG enrichment analysis of common, up-regulated, and down-regulated DEGs in each treatment comparison. Common-1 indicates the common up-regulated DEGs in R24 vs. R0, R36 vs. R0, and R72 vs. R0. Common-2 is the common down-regulated DEGs in S24 vs. S0, S36 vs. S0, and S72 vs. S0. **(B)** A big Venn diagram of DEGs for up-regulated DEGs in Rs vs. R0 and down-regulated DEGs in Ss vs. S0.

When resistant peanut A165 leaves were infected by HA4-1, a series of genes were induced. These genes were thought to be beneficial to activate immune responses. There are 3,943, 6,482, and 3,779 up-regulated DEGs in R24 vs. R0, R36 vs. R0, and R72 vs. R0, respectively (Table 1; Supplementary Table S3). The number of up-regulated DEGs in R36 vs. R0 is markedly more than those at the other two time points. KEGG enrichment showed that these DEGs were mainly involved in plant hormone signal transduction, amino sugar and nucleotide sugar metabolism, and MAPK signaling pathway (Figure 3A; Supplementary Table S4). Previously, our qRT-PCR results demonstrated that the expression of known resistant genes *AhRLK1*, *AhRRS5*, and *AhGLK1b* reached the highest level in resistant peanut leaves inculcated by HA4-1 at 72 h. Therefore, we paid more attention to the up-regulated DEGs in R72 vs. R0. For these DEGs, flavonoid biosynthesis, plant-pathogen interaction and MAPK signaling pathway are the significantly enriched KEGG pathways (Figure 3A; Supplementary Table S4). To further narrow down the scope of vital candidate resistance response genes, we performed the Venn analysis to find the common DEGs in the aforementioned up-regulated genes obtained 818 DEGs (Figure 3B; Supplementary Table S5 Sheet1). KEGG enrichment analysis showed that these common DEGs were mainly enriched in Linoleic acid metabolism, phenylpropanoid biosynthesis, and plant-pathogen interaction (Figure 3A; Supplementary Table S4).

Similarly, when inoculated by HA4-1, the expression of many genes was reduced in the susceptible peanut A281 leaves. These genes were therefore thought to negatively regulated susceptibility, that is, they were also resistance response genes. There are 1,394, 7,463, and 7,341 down-regulated DEGs in S24 vs. S0, S36 vs. S0, and S72 vs. S0, respectively (Figure 3A; Supplementary Table S4). The significantly enriched KEGG pathways involved in the down-regulated DEGs of S36 vs. S0 and S72 vs. S0 were similar, such as plant hormone signal transduction, photosynthesis, as well as glyoxylate and dicarboxylate metabolism (Figure 3A; Supplementary Table S4). Venn analysis revealed that 612 DEGs were common in these comparison groups (Figure 3B; Supplementary Table S5 Sheet2). Plant hormone signal transduction, phenylpropanoid biosynthesis, and glycerophospholipid metabolism are the significantly enriched KEGG pathways (Figure 3A; Supplementary Table S4).

Further Venn analysis showed that a total of 78 DEGs were common between up-regulated DEGs in Rs vs. R0 and down-regulated DEGs in Ss vs. S0 (Figure 3B; Supplementary Table S5 Sheet3). The 78 common DEGs mainly involved in calcium transport and localization (Supplementary Table S5 Sheet3). These genes need to be analyzed in-depth for their molecular function in peanut response to *R. solanacearum*.

#### Susceptibility-response DEGs

**Figure 4:**
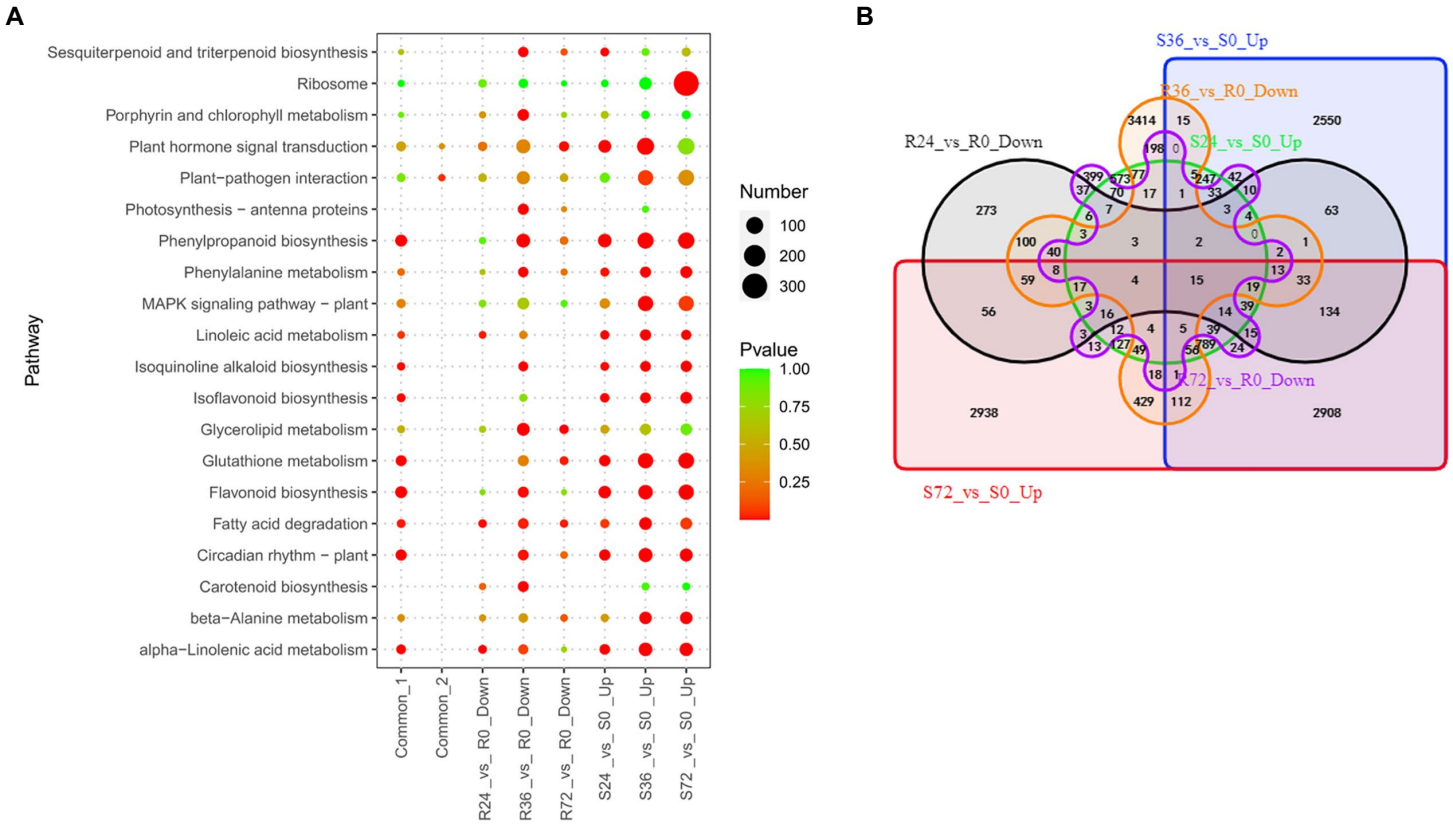
Identification of susceptibility-response DEGs in R24 vs. R0, R36 vs. R0, R72 vs. R0, S24 vs. S0, S36 vs. S0, and S72 vs. S0. **(A)** KEGG enrichment analysis of common, up-regulated, and down-regulated DEGs in each treatment comparison. Common-1 indicates the common up-regulated DEGs in S24 vs. S0, S36 vs. S0, and S72 vs. S0. Common-2 is the common down-regulated DEGs in R24 vs. R0, R36 vs. R0, and R72 vs. R0. **(B)** A big Venn diagram of DEGs for up-regulated DEGs in Ss vs. S0 and down-regulated DEGs in Rs vs. R0.

Once the *R. solanacearum* enters into the host, the virulence factors will attack the immune system. At the same time, the expression of many genes will be increased in susceptible plants. These genes are a set of susceptibility-response genes. To reveal the susceptibility-response DEGs, we analyzed the transcriptome changes of susceptible peanut A281 leaves infected by HA4-1. There are 2,259, 7,194, and 7,972 up-regulated DEGs in S24 vs. S0, S36 vs. S0, and S72 vs. S0, respectively (Table 1; Supplementary Table S3). The number of up-regulated DEGs in S24 vs. S0 was greatly less than those in the other two comparison groups. There were many up-regulated DEGs involved in flavonoid biosynthesis, phenylpropanoid biosynthesis, and plant hormone signal transduction in S24 vs. S0 (Figure 4A; Supplementary Table S6). A number of DEGs enriched in Flavonoid biosynthesis, MAPK signaling pathway, and plant hormone signal transduction in the up-regulated DEGs of S36 vs. S0 (Figure 4A; Supplementary Table S6). In up-regulated DEGs of S72 vs. S0, ribosome, flavonoid biosynthesis, and phenylpropanoid biosynthesis were the significantly enrichment KEEG pathways (Figure 4A; Supplementary Table S6). The Flavonoid biosynthesis is conspicuous at all three time points which indicate it is a significant pathway in the susceptible peanut response to *R. solanacearum*. Venn analysis showed that there were 976 common DEGs in up-regulated DEGs among the three comparison groups (Figure 4B; Supplementary Table S7 Sheet1). KEGG enrichment showed that these 976 common DEGs were mainly involved in flavonoid biosynthesis, glycolysis, circadian rhythm, and phenylpropanoid biosynthesis (Figure 4A; Supplementary Table S6).

On the other hand, when the resistant peanut was infected by HA4-1, the expression of some genes was also suppressed. These genes were another set of susceptibility-response genes. There were 1,022, 4,720 and 1,068 down-regulated DEGs in R24 vs. R0, R36 vs. R0, and R72 vs. R0, respectively (Table 1; Supplementary Table S3). Ribosome biogenesis, photosynthesis, and glycerolipid metabolism were the significantly enrichment KEGG pathways among these down-regulated DEGs. Eighty-seven common DEGs were identified by Venn analysis (Figure 4B; Supplementary Table S7 Sheet2), and these genes were significantly enriched in the pathways of terpenoid backbone biosynthesis, one carbon pool by folate, and plant-pathogen interaction (Figure 4A; Supplementary Table S6).

Venn analysis showed that only 15 DEGs were common between the two sets of susceptibility-response genes (Figure 4B; Supplementary Table S7 Sheet3), and they were mainly involved in transmembrane transport (Supplementary Table S7 Sheet3). The biological function of these genes in the peanut response to *R. solanacearum* needs more experiments to demonstrate in future studies.

### Genes influenced by PAMPs were revealed by analyzing the response to *ΔhrpB* mutant strain

To combat pathogens, plants have evolved a multi-layered innate immune system to reject or attenuate infection, the so-called pattern-triggered immunity (PTI) and effector-triggered immunity (ETI). Once *R. solanacearum* infected peanut leaves, PTI is first activated, and then T3Es function. The ETI response is activated by the interaction between T3Es and R proteins, which is the strongest immune pathway in plant-pathogen interaction. Both PTI and ETI can induce transcriptional reprogramming of different sets of defense-related genes to confer resistance. In the present study, wild-type *R. solanacearum* HA4-1 not only possess various PAMPs but also secrete all its T3Es, when it infected peanuts, both PTI and possible ETI occur. While its *ΔhrpB* mutant, which was unable to secrete T3Es, infected peanuts, only PTI occur. In order to better understand how these two immune responses occur in peanut leaves infected with *R. solanacearum*, as well as screen out the relevant genes and pathways, we also analyzed the different transcriptome changes of peanut leaves infected by wild-type and *ΔhrpB* mutant *R. solanacearum* strains. Next, we will sort out the PAMPs and T3Es caused DEGs, and analyze them in detail. Similarly, we will also analyze these DEGs in the context of resistant and susceptible peanuts, respectively.

#### PAMPs induced resistance-response DEGs

**Figure 5:**
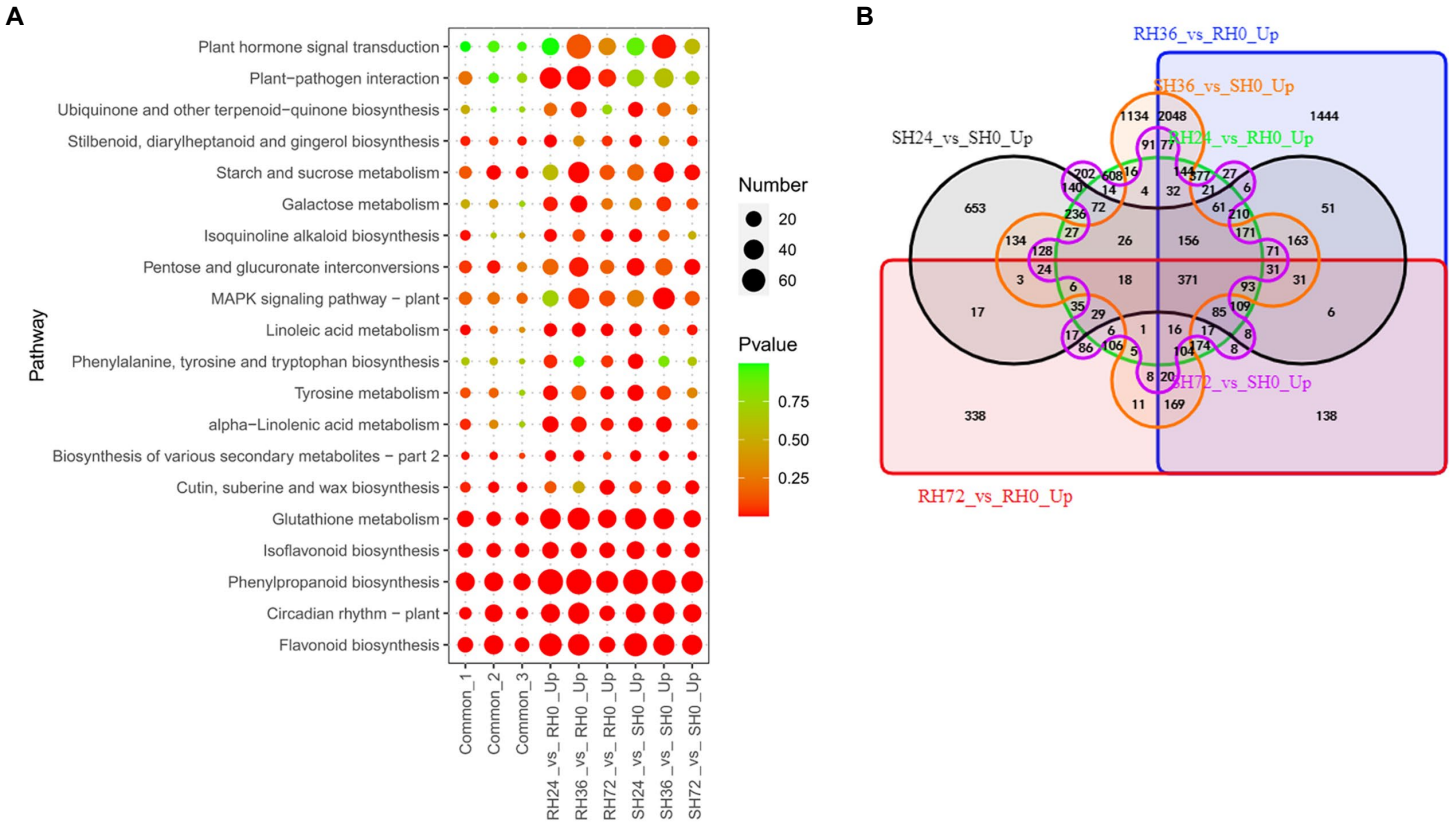
Identification of PAMPs induced resistance-response DEGs in RH24 vs. RH0, RH36 vs. RH0, RH72 vs. RH0, SH24 vs. SH0, SH36 vs. SH0, and SH72 vs. SH0. **(A)** KEGG enrichment analysis of common and up-regulated DEGs in each comparison group. Common-1 indicates the common up-regulated DEGs in RH24 vs. RH0, RH36 vs. RH0, and RH72 vs. RH0. Common-2 is the common up-regulated DEGs in SH24 vs. SH0, SH36 vs. SH0, and SH72 vs. SH0. Common-3 is the common up-regulated DEGs between RHs vs. RH0 and SHs vs. SH0. **(B)** A big Venn diagram of DEGs for up-regulated DEGs between RHs vs. RH0 and SHs vs. SH0.

Both resistant and susceptible peanuts inoculated with *ΔhrpB* mutants did not develop disease, which is partly because the pathogen lacks the crucial pathogenic factor T3Es and partly because PAMPs stimulate basal immune response of the peanuts. When resistant and susceptible peanuts were infected by *ΔhrpB* mutant, the expression of a number of genes was significantly up-regulated. Up-regulation of these genes was thought to be induced by PAMPs and ultimately lead to the disease-resistant phenotype. To reveal these DEGs, we analyzed the transcriptome changes of peanut leaves infected by *ΔhrpB* mutant. There are 3,350, 6,439, 2,090, 3,188, 5,333 and 1,873 up-regulated DEGs in RH24 vs. RH0, RH36 vs. RH0, RH72 vs. RH0, SH24 vs. SH0, SH36 vs. SH0, and SH72 vs. SH0, respectively (Table 1; Supplementary Table S3). These DEGs were mainly enriched in flavonoid biosynthesis, phenylpropanoid biosynthesis, and glutathione metabolism in the six comparison groups (Figure 5A; Supplementary Table S8). By Venn analysis, we found 969 common up-regulated DEGs among RH24 vs. RH0, RH36 vs. RH0 and RH72 vs. RH0 (Figure 5B; Supplementary Table S9 Sheet1), which were significantly enriched in phenylpropanoid biosynthesis, glutathione metabolism, and flavonoid biosynthesis (Figure 5A; Supplementary Table S8). There were 825 common up-regulated DEGs among SH24 vs. SH0, SH36 vs. SH0, and SH72 vs. SH0 (Figure 5B; Supplementary Table S9 Sheet2). KEGG enrichment analysis of the 825 common DEGs showed that they were mainly enriched in flavonoid biosynthesis, circadian rhythm, and phenylpropanoid biosynthesis. Further analysis showed that there were 371 common up-regulated DEGs between RHs vs. RH0 and SHs vs. SH0 (Figure 5B; Supplementary Table S9 Sheet3). They were mainly enriched in phenylpropanoid biosynthesis, flavonoid biosynthesis, and isoflavonoid biosynthesis (Figure 5A; Supplementary Table S8).

#### PAMPs induced susceptibility-response DEGs

**Figure 6:**
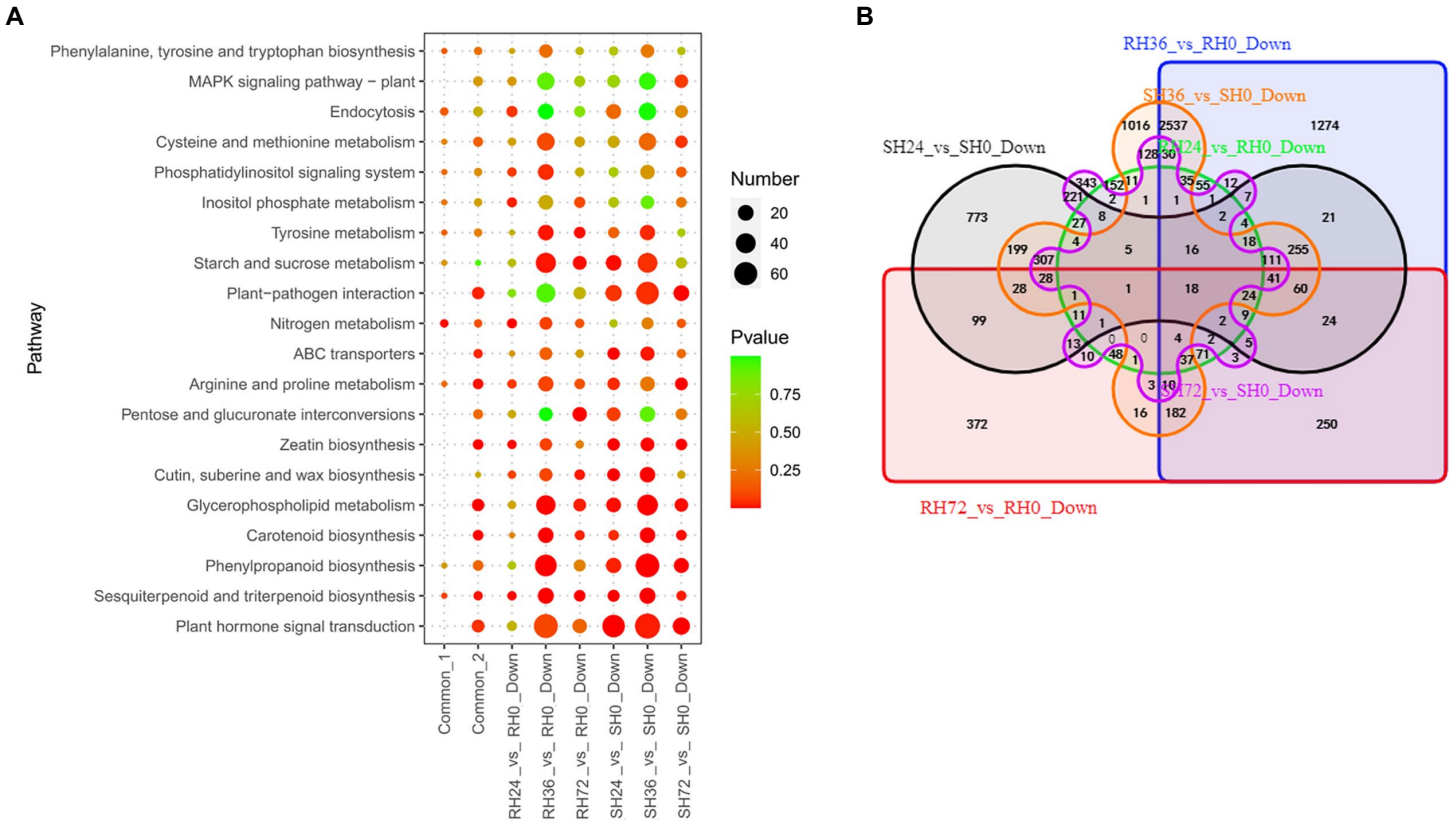
Identification of PAMPs induced susceptibility-response DEGs in RH24 vs. RH0, RH36 vs. RH0, RH72 vs. RH0, SH24 vs. SH0, SH36 vs. SH0, and SH72 vs. SH0. **(A)** KEGG enrichment analysis of common and down-regulated DEGs in each comparison group. Common-1 and Common-2 indicate the common up-regulated DEGs in RHs vs. RH0 and SHs vs. SH0, respectively. **(B)** A big Venn diagram of DEGs for down-regulated DEGs in RHs vs. RH0 and SHs vs. SH0.

When infected by *ΔhrpB* mutant strain which lacks all the T3Es, peanuts did not show wilt symptoms, but some genes were differently expressed. For instance, the expression of some genes was suppressed. Down-regulation of these genes was thought to be caused by PAMPs and other virulence factors. We found many genes were down-regulated in the samples infected by *ΔhrpB* mutant. For example, there are 572, 5,121, 1,374, 2,343, 5,128 and 1,336 down-regulated DEGs in RH24 vs. RH0, RH36 vs. RH0, RH72 vs. RH0, SH24 vs. SH0, SH36 vs. SH0 and SH72 vs. SH0, respectively (Table 1; Supplementary Table S3). They were mainly enriched in nitrogen metabolism, plant hormone signal transduction, and plant-pathogen interaction (Figure 6A; Supplementary Table S10). By Venn analysis, we found there were 167 common down-regulated DEGs among RH24 vs. RH0, RH36 vs. RH0, and RH72 vs. RH0 (Figure 6B; Supplementary Table S11 Sheet2). There were 527 common down-regulated DEGs among SH24 vs. SH0, SH36 vs. SH0, and SH72 vs. SH0 (Figure 6B; Supplementary Table S11 Sheet2). Plant hormone signal transduction, plant−pathogen interaction, and glycerophospholipid metabolism were the significantly enrichment pathways (Figure 6A; Supplementary Table S10). Further analysis showed that there were 18 common down-regulated DEGs between RHs vs. RH0 and SHs vs. SH0 (Figure 6B; Supplementary Table S11 Sheet3). GO enrichment analysis demonstrated that they were mainly involved in anion transport (Supplementary Table S11 Sheet3).

### Genes influenced by T3Es were revealed by comparing HA4-1 with *ΔhrpB* mutant inoculation

In the plant response to *R. solanacearum*, a series of T3Es, including avirulence and virulence T3Es, is injected into plant cells. The interaction between avirulence T3Es and R proteins induces the resistant response, and the virulence T3Es activate the susceptibility response. The wild-type *R. solanacearum* not only secrete all its T3Es but also possess various PAMPs and other virulence factors. When it infected peanuts, all factors function. While its *ΔhrpB* mutant, which was unable to secrete T3Es, infected peanuts, only PAMPs and other virulence factors function. The only difference between the two bacteria is the presence of T3Es or not, so the DEGs influenced by T3Es can be obtained by comparing HA4-1 with *ΔhrpB* mutant inoculation (Rs vs. RHs and Ss vs. SHs). When the resistance peanut A165 was infected by *R. solanacearum*, potential R proteins that recognize potential avirulence T3Es could directly or indirectly lead to the disease resistance response. In this case, avirulence T3Es elicit the expression of a series of genes associated with defense response and suppress the expression of genes associated with susceptibility response. Conversely, when *R. solanacearum* infected the susceptible peanut A281, the susceptibility response was triggered by the virulence T3Es, which induced the expression of genes associated with the susceptibility response and suppressed the expression of genes associated with defense response.

#### T3Es induced resistance-response DEGs

**Figure 7:**
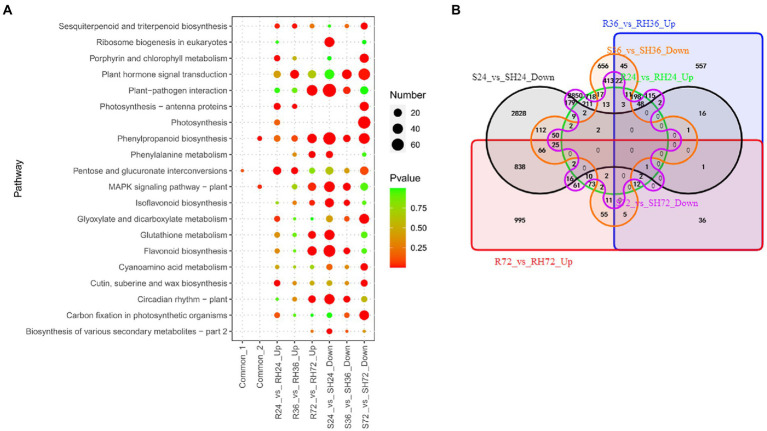
Identification of T3Es induced resistance-response DEGs in R24 vs. RH24, R36 vs. RH36, R72 vs. RH72, S24 vs. SH24, S36 vs. SH36, and S72 vs. SH72. **(A)** KEGG enrichment analysis of common, up-regulated, and down-regulated DEGs in each treatment comparison. Common-1 indicates the common up-regulated DEGs in R24 vs. RH24, R36 vs. RH36, and R72 vs. RH72. Common-2 is the common down-regulated DEGs in S24 vs. SH24, S36 vs. SH36, and S72 vs. SH72. **(B)** A big Venn diagram of DEGs for up-regulated DEGs in Rs vs. RHs and down-regulated DEGs in Ss vs. SHs.

When HA4-1 infected resistant peanut A165 leaves, avirulence T3Es will interact with potential R proteins and the downstream defense response will be activated. Because *ΔhrpB* mutant only uses PAMPs to activate responses in the infected peanut leaves, the up-regulated DEGs in Rs vs. RH were resistance-response DEGs induced by T3Es. There are 1,338, 1,057, and 2,213 up-regulated DEGs in R24 vs. RH24, R36 vs. RH36, and R72 vs. RH72, respectively (Table 1; Supplementary Table S3). These DEGs were mainly enriched in plant hormone signal transduction, plant-pathogen interaction, and MAPK signaling pathway in R36 vs. RH36, and R72 vs. RH72 (Figure 7A; Supplementary Table S12). There were 15 common up-regulated DEGs among R24 vs. RH24, R36 vs. RH36, and R72 vs. RH72 (Figure 7B; Supplementary Table S13 Sheet1). KEGG enrichment analysis of the 15 common DEGs showed that they were mainly enriched in pentose and glucuronate interconversions (Figure 7A; Supplementary Table S12).

Comparing HA4-1 with *ΔhrpB* mutant inoculation, the down-regulated DEGs in susceptible peanut A281 leaves also may be the T3Es induced resistant-response genes. There were 4,152, 1,513, and 4,038 down-regulated DEGs in S24 vs. SH24, S36 vs. SH36, and S72 vs. SH72, respectively (Table 1; Supplementary Table S3). KEGG enrichment analysis revealed that plant-pathogen interaction, MAPK signaling pathway, and plant hormone signal transduction were the mainly enrichment pathways (Figure 7A; Supplementary Table S12). By Venn analysis, we found there were 77 common down-regulated DEGs among S24 vs. SH24, S36 vs. SH36, and S72 vs. SH72 (Figure 7B; Supplementary Table S13 Sheet2). These common DEGs were mainly involved in phenylpropanoid biosynthesis and MAPK signaling pathway (Figure 7A; Supplementary Table S12). Further analysis showed that there were no common DEGs between up-regulated DEGs in Rs vs. RHs and down-regulated DEGs in Ss vs. SHs (Figure 7B).

#### T3Es induced susceptibility-response DEGs

**Figure 8:**
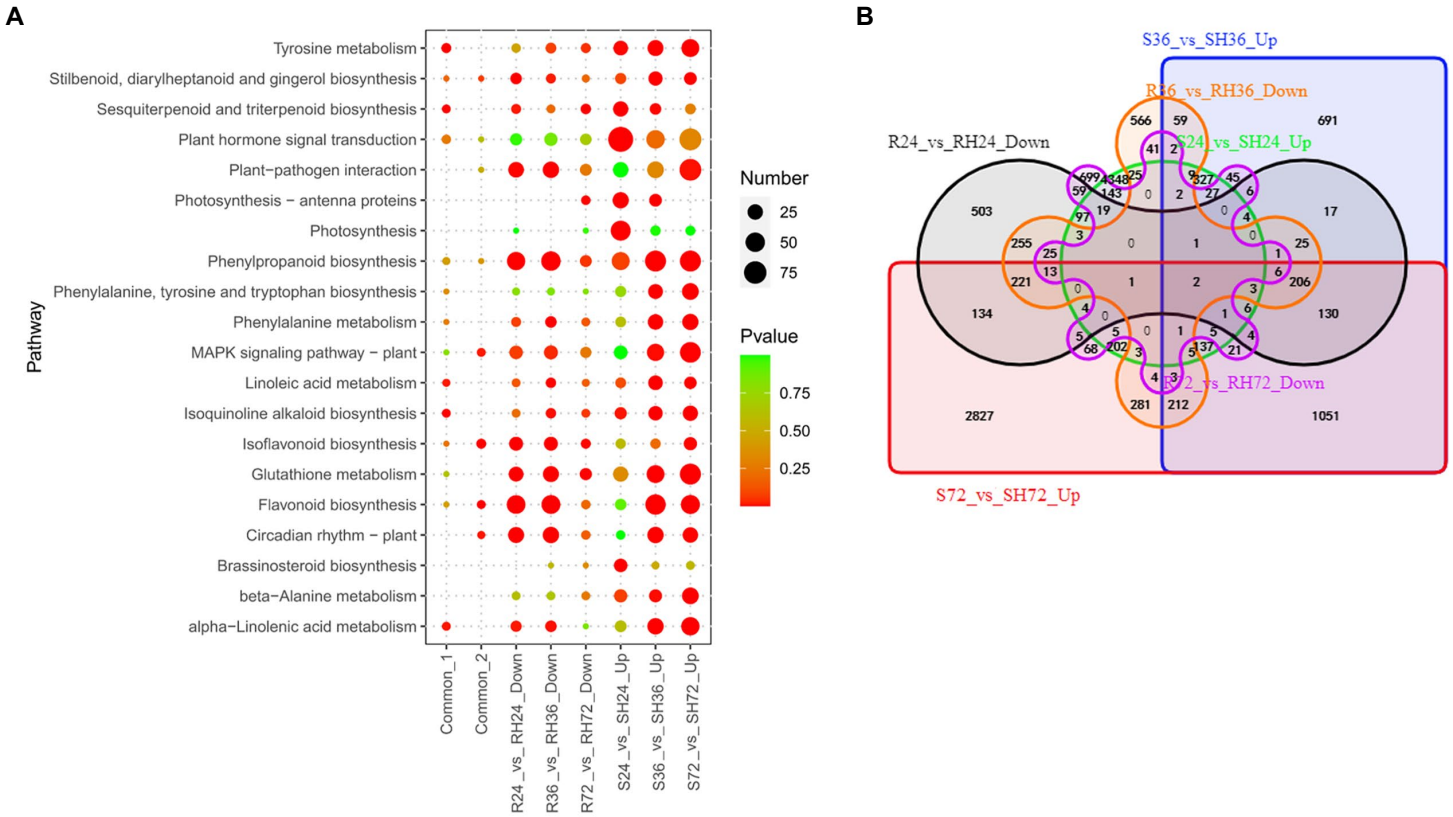
Identification of T3Es induced susceptibility-response DEGs in R24 vs. RH24, R36 vs. RH36, R72 vs. RH72, S24 vs. SH24, S36 vs. SH36, and S72 vs. SH72. **(A)** KEGG enrichment analysis of common, up-regulated, and down-regulated DEGs in each treatment comparison. Common-1 indicates the common up-regulated DEGs in S24 vs. SH24, S36 vs. SH36, and S72 vs. SH72. Common-2 is the common down-regulated DEGs in R24 vs. RH24, R36 vs. RH36, and R72 vs. RH72. **(B)** A big Venn diagram of DEGs for up-regulated DEGs in Ss vs. SHs and down-regulated DEGs in Rs vs. RHs.

Effectors may influence the health of plants by speeding up certain physiological processes that are detrimental to plants. The genes involved in these physiological processes can be regarded as another set of susceptibility-response genes. In this study, T3Es induce the expression of genes in susceptible peanuts to accelerate the course of the disease, and it is the opposite situation in resistant peanuts. We analyzed the up-regulated genes in S24 vs. SH24 and S36 vs. SH36, and revealed 5,380, 3,009, and 5,561 up-regulated DEGs in the three comparison groups, respectively (Table 1; Supplementary Table S3). These DEGs were mainly enriched in plant-pathogen interaction, plant hormone signal transduction, and MAPK signaling pathway (Figure 8A; Supplementary Table S14). There were 160 common up-regulated DEGs among S24 vs. SH24, S36 vs. SH36, and S72 vs. SH72 (Figure 8B; Supplementary Table S15 Sheet1). KEGG enrichment analysis of the 160 common DEGs showed that they significantly enriched in tyrosine metabolism (Figure 8A; Supplementary Table S14).

These down-regulated DEGs may be T3Es induced susceptibility-response genes in R24 vs. RH24, R36 vs. RH36, and R72 vs. RH72. There are 1,751, 1,975 and 1,209 down-regulated DEGs in the three comparison groups, respectively (Table 1; Supplementary Table S3). These DEGs were mainly enriched in plant-pathogen interaction and MAPK signaling pathway in R24 vs. RH24 and R36 vs. RH36 (Figure 8A; Supplementary Table S14). There were 49 common down-regulated DEGs among R24 vs. RH24, R36 vs. RH36, and R72 vs. RH72 (Figure 8B; Supplementary Table S15 Sheet2). KEGG enrichment analysis revealed that MAPK signaling pathway was significantly enriched (Figure 8A; Supplementary Table S14). There were only two common DEGs between up-regulated DEGs in Ss vs. SHs and down-regulated DEGs in Rs vs. RHs, which, respectively, function annotated as Hevamine-A (*AH19G35340*) and metallothiol transferase FosB-like (*AH04G02610*; Figure 8B; Supplementary Table S15 Sheet3).

### Validation of transcriptomics data by quantitative real-time RT-PCR

**Figure 9:**
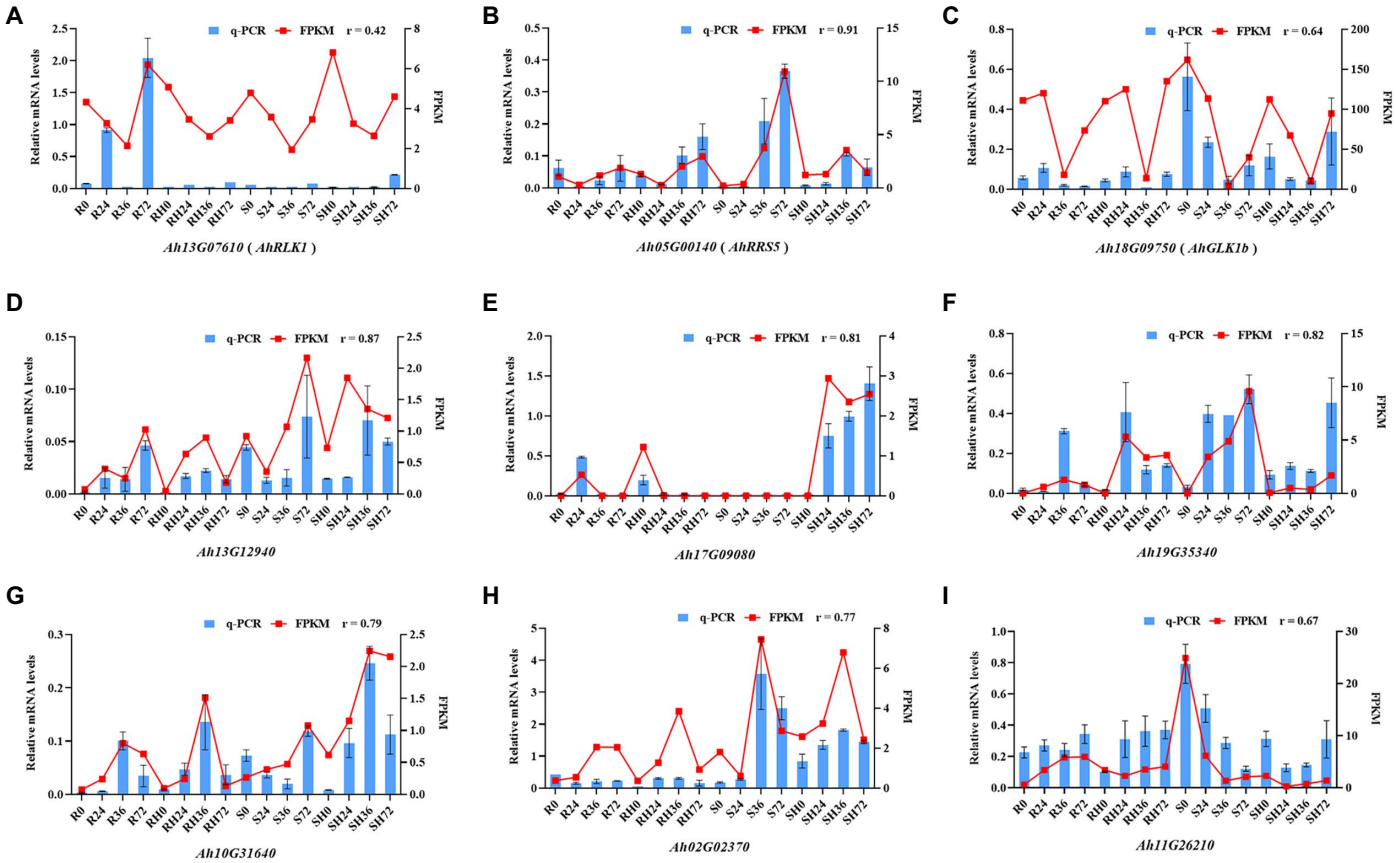
Coefficient analysis of the consistency between qRT-PCR data and RNA-Seq results. Error bars indicate ± SE. *r* represents the correlation coefficient.

To confirm the accuracy of the RNA-seq data, the expression of three BW resistance-related genes *AhRLK1*, *AhRRS5,* and *AhGLKb* at four time points after RSI was analyzed by qRT-PCR with three biological replicates. The correlation coefficient between qRT-PCR data and RNA-Seq results were 0.42, 0.91, and 0.64 for *AhRLK1*, *AhRRS5,* and *AhGLKb*, respectively (Figures 9A–C). The low relation for *AhRLK1* was possibly caused by diverse sensitivities and algorithms between these two detection methods. To further verify the RNA-Seq results, we randomly selected 6 DEGs to detect mRNA expression by qRT-PCR. The qRT-PCR data for these genes were significantly correlated with the RNA-Seq results (a mean correlation coefficient of 0.79; Figures 9D–I). These results indicated that the RNA-seq results in the present study were reliable.

## Discussion

A fast and reliable bioassay for disease phenotype evaluation is very important to reveal the molecular mechanism of the peanut response to *R. solanacearum*. To screen BW-resistant and-susceptible cultivated peanuts, we developed a simple cutting leaf method to evaluate the disease phenotype (Tan et al., 2022). In the present study, we confirmed the significantly different disease symptoms in leaves after inoculation with HA4-1 to investigate if the method is applicable for resistant A165 and susceptible A281. The wilting symptoms gradually appeared on the whole plant after the four leaflets were cut with the HA4-1-stained scissors in A281 (Figure 1A). The disease symptom of A281 was significantly higher than A165 through the statistical analysis of disease index and survival ratio (Figures 1B,C). A165 inhibited the proliferation of *R. solanacearum* and displayed a lower level of electrolyte leakage compared with A281 (Figures 1D,E). The same BW phenotype also appeared in A165 and A281 when the frequently-used root inoculation method was performed (Supplementary Figure S3), which confirmed the feasibility of the cutting leaf method. Through these results, we confirmed that A165 was a clear BW resistance cultivar compared with A281. Compared with the root inoculation method, the cutting leaf method is easy to operate and investigate phenotype, which is more useful to better understand the interaction between peanuts and *R. solanacearum* and thus should be more used. In the following studies, the cutting leaf method will be employed to elucidate the molecular mechanism of the peanut response to *R. solanacearum*.

Few studies have been performed to characterize the interaction between peanuts and *R. solanacearum* at molecular level. Transcriptional reprogramming in root tissue following soil-drenching with *R. solanacearum* has been previously reported in peanuts (Chen et al., 2014). No information is available about RNA-seq analysis of transcriptional changes in aerial parts of peanuts treated with *R. solanacearum*. PTI and ETI are the main molecular events occurring in plants with RSI. To separately uncover the genes involved in PTI and ETI, we employed the *ΔhrpB* mutant strain of HA4-1, which cannot deliver T3Es into plant cells to induce ETI. A comparative transcriptomic analysis was performed in A165 and A281 leaf tissue following the cutting leaf with HA4-1 and *ΔhrpB* mutant strain to investigate the molecular mechanism underlying the peanut-*R. solanacearum* interaction. A total of 125,978 DEGs were identified according to the screen criteria (Table 1; Supplementary Table S3). To elucidate the molecular mechanism of the peanut response to *R. solanacearum*, we classified these DEGs into resistance-response genes, susceptibility-response genes, PAMPs induced resistance-response genes, PAMPs induced susceptibility-response genes, T3Es induced resistance-response genes, and T3Es induced susceptibility-response genes. GO and KEGG enrichment analysis clearly implicated several pathways, including the calcium signaling pathway, the plant-pathogen interaction, the plant hormone signal transduction, and the MAPK signaling pathway, which may provide additional candidate genes for understanding the molecular mechanism of the peanut response to *R. solanacearum*.

Several early signaling events are induced during plant-pathogen interaction, such as calcium flux, the activation of mitogen-activated protein kinases (MAPKs), the production of reactive oxygen species (ROS), and the induction of plant hormone biosynthesis. The influx of Ca^2+^ from outside the cell is among the earliest event upon pathogen attack in the plant cells, which is essential for plant immunity (Du et al., 2009; He et al., 2019). The Ca^2+^ signaling is transduced *via* sensors including calmodulin-like (CML) proteins, calcium-dependent protein kinases (CDPKs), and calcineurin B-like proteins (CBLs). It had been demonstrated that calcium sensors played major roles in the regulation of plant immunity against pathogen attacks. Upon binding to Ca^2+^, calmodulin (CaM) interacts with numerous target proteins and modulates their activity, which have been found to be targeted by T3Es from pathogens to repress PTI in the host plants (Zheng et al., 2018; Naveed et al., 2019). Although CMLs are structurally similar to CaMs, the number of CMLs in plants is far more than CaMs (Boonburapong and Buaboocha, 2007; Li et al., 2019). Different CMLs have diverse functions in plant immunity, CML8, CML9, and CML24 act as positive regulators against *Pseudomonas syringae pv. Tomato* DC3000 in *Arabidopsis thaliana*, while CML46 and CML47 are identified as negative regulators (Zhu et al., 2017a,b). In pepper immunity against *R. solanacearum*, CML13 functions as a positive regulator that forms a positive feedback loop with bZIP63 (Shen et al., 2020). Excepting CMLs, CDPKs are also important positive regulators in plant immunity. CDPKs not only serve as convergence points of signaling triggered by most PAMPs (Boudsocq and Sheen, 2013), but also are versatile activators of signaling components including the Nucleotide-Binding Leucine-Rich Repeat Receptors (NLRs; Liu et al., 2017), and transcription factors such as WRKY proteins (Zhou et al., 2020). During pepper response to RSI, WRKY27b is phosphorylated by CDPK29 and acts as a transcriptional activator of WRKY40 to mediate the immunity response (Yang et al., 2022). In the present study, many CML and CDPK genes were identified as DEGs in different comparison groups, and some of them were significantly enriched in plant-pathogen interaction (Supplementary Table S16 Sheet1). When peanut leaves were inoculated by *R. solanacearum*, Ca^2+^ signaling pathway was activated, which would be decoded and transmitted downstream by CML and CDPK proteins. In order to survive the challenges imposed by *R. solanacearum*, a series of specific cellular and physiological responses will be activated by CMLs and CDPKs relaying or decoding the encoded Ca^2+^ signals. In response to *R. solanacearum*, CMLs and CDPKs activate the downstream resistance genes to induce defense reaction in the resistant peanut A165, while they are likely to serve as negative regulators to promote susceptibility response in the susceptible peanut A281. Interestingly, we also found that WRKY33 enriched in plant-pathogen interaction was consistently highly expressed in the different comparison groups (Supplementary Table S15 Sheet1). Calcium sensors might promote the binding of WRKY33 to the promoters of resistance- or susceptibility-related genes to enhance their transcriptional reprogramming during peanut response to *R. solanacearum*. The mechanism of CMLs or CDPKs-WRKY transcription factor module mediated peanut response to *R. solanacearum* needs more detailed experimental data to fully understand. Overall, our results suggested that calcium sensors and WRKY transcription factors acted together as positive or negative regulators in peanut response to *R. solanacearum*.

Plant MAPK cascades, as another early signaling event, play important roles in regulating plant responses to pathogen infection (Meng and Zhang, 2013; Thulasi Devendrakumar et al., 2018). In general, plant MAPK cascades comprise MAPK Kinase Kinases (MAPKKKs/MEKKs), MAPK Kinases (MAPKKs/MKKs), and MAPKs/MPKs, which sequentially activate each other by phosphorylation. An active MPK can ultimately phosphorylate the downstream substrates and induce appropriate cellular responses. Upon PAMP perception, two cascades, MEKK3/5-MKK4/5-MPK3/6 and MEKK1-MKK1/2-MPK4, are rapidly activated. These rapid but transient activations are critical for the transcriptional reprogramming that is the characteristic of PTI (Lang and Colcombet, 2020). Active MPK3/6, with a delayed and sustained kinetic response, have also been found during ETI in different plant species. Some other MAPK components have been demonstrated to employ specific contributions to the ETI responses. As the downstream components of MAPK cascades, WRKY-type transcription factors are phosphorylated by MPK to activate, which constitute a complex defense response network as both positive and negative regulators in plant immunity (Pandey and Somssich, 2009). MPK3, MPK4, and MPK6 were demonstrated to be activated by pathogen elicitors in *Arabidopsis* and play a crucial role in innate immune responses (Takahashi et al., 2007; Berriri et al., 2012; Eschen-Lippold et al., 2016). GmMPK4 negatively regulated the defense responses to downy mildew and mosaic virus in soybean (Liu et al., 2011). CaWRKY27 and CaWRKY40 took a positive role in resistance to *R. solanacearum* in pepper and tobacco, respectively, while AtWRKY27 played a negative role in response to *R. solanacearum* in *A. thaliana* (Mukhtar et al., 2008; Dang et al., 2013, 2014). In our study, MAPK signaling pathway was the significantly enrichment KEGG pathway, which was consistent with the previous report in transcriptomic analysis of peanut roots with RSI (Chen et al., 2014). We found that MEKK1 started to significantly up-regulate expression at 36 hpi in Rs vs. R0, RHs vs. RH0, Ss vs. S0, and SHs vs. SH0, while it was at 72 hpi in Rs vs. RHs and Ss vs. SHs (Supplementary Table S16 Sheet2). The MKK2 and MPK3 also displayed the similar expression pattern with MEKK1. It suggested that the MEKK1-MKK2-MPK3 cascade was rapidly activated upon PAMP perception in peanut leaves, then it was later activated by T3Es. The DEGs encoding WRKYs showed different expression patterns in the A165 and A281 leaves with RSI. For instance, WRKY22 was up-regulated in A281, and WRKY33 was up-regulated in A165 and A281 (Supplementary Table S16 Sheet2). It could be deduced that the WRKYs both took positively and negatively in peanut response to *R. solanacearum*. The exact roles of the MEKK1-MKK2-MPK3 cascade and WRKYs in peanut response to *R. solanacearum* still need further study.

Plant hormones are very important signaling molecules involved in the regulation of plant-pathogen interactions (Shigenaga and Argueso, 2016). Salicylic acid, jasmonic acid, ethylene, abscisic acid, gibberellic acid, cytokinin, auxin, and brassinosteroids are the known plant hormones associated with plant-pathogen interaction, and they act as positive or negative regulators in plant immunity. Auxin (AUX), a key plant hormone, also has both positive and negative effects on plant defense (Kazan and Manners, 2009; Fu and Wang, 2011; Ludwig-Müller, 2015). During plant-pathogen interaction, auxin functions mainly to promote susceptibility, and many pathogens produce auxin to promote disease, including *R. solanacearum* (Valls et al., 2006; Shigenaga and Argueso, 2016). Suppression of auxin signaling has been reported to be associated with plant resistance to vascular wilt pathogens. For example, the tomato auxin transport mutant was resistant to *R. solanacearum* (French et al., 2018). Indole-3-acetic acid (IAA), phenylacetic acid (PAA) and 4-chloro-indole-3-acetic acid (4-Cl-IAA) are naturally occurring auxins in plants, of which IAA is the most prominent and best studied auxin (Casanova-Sáez et al., 2021). In the context of low concentration IAA, the expression of auxin-responsive genes is inhibited or eliminated through the interaction of dominant AUX/IAA repressors with auxin response factor (ARF) activators on the promoter of these genes (Hagen, 2015). When the concentration of IAA increases significantly by environment stimuli, the AUX/IAA transcriptional repressors will be ubiquitylated and degraded by the 26S proteasome, resulting in activation of auxin response factors and expression of auxin-responsive genes. Previous studies showed that pathogens have evolved to target the auxin pathway to regulate its synthesis and signaling in order to enhance virulence. In Arabidopsis response to *Pseudomonas syringae*, the type III effector AvrRpt2 manipulates plant auxin signaling by promoting the degradation of AUX/IAA transcriptional repressors, thereby activating the ARFs and increasing the expression of auxin-responsive genes to promote pathogen growth and disease development (Chen et al., 2007; Cui et al., 2013). Recently, it was demonstrated that the effector protein Naked1 from *Ustilago maydis* increased the auxin signaling by preventing the recruitment of AUX/IAA transcriptional repressors to promote maize susceptibility to (hemi) biotrophic pathogens (Navarrete et al., 2022). In the present study, many DEGs were enriched in plant hormone signal transduction, including many auxin-responsive genes (Supplementary Table S16 Sheet3). Most of auxin-responsive genes were significantly up-regulated expression in S24 vs. SH24 and down-regulated expression in RHs vs. RH0 and SHs vs. SH0, a few were up-regulated expression in Rs vs. R0, Rs vs. RHs and Ss vs. S0. It suggested that T3Es from *R. solanacearum* also might enhance auxin signaling by inhibiting the AUX/IAA transcriptional repressors to increase susceptibility to pathogens in susceptible peanut A281. In resistant peanut A165 response to *R. solanacearum*, it was likely that auxin signaling was repressed by inhibiting the expression of some auxin-responsive genes to increase peanut resistance to pathogens. How auxin modulates the peanut response to *R. solanacearum* still needs more experiments to demonstrate.

## Conclusion

**Figure 10:**
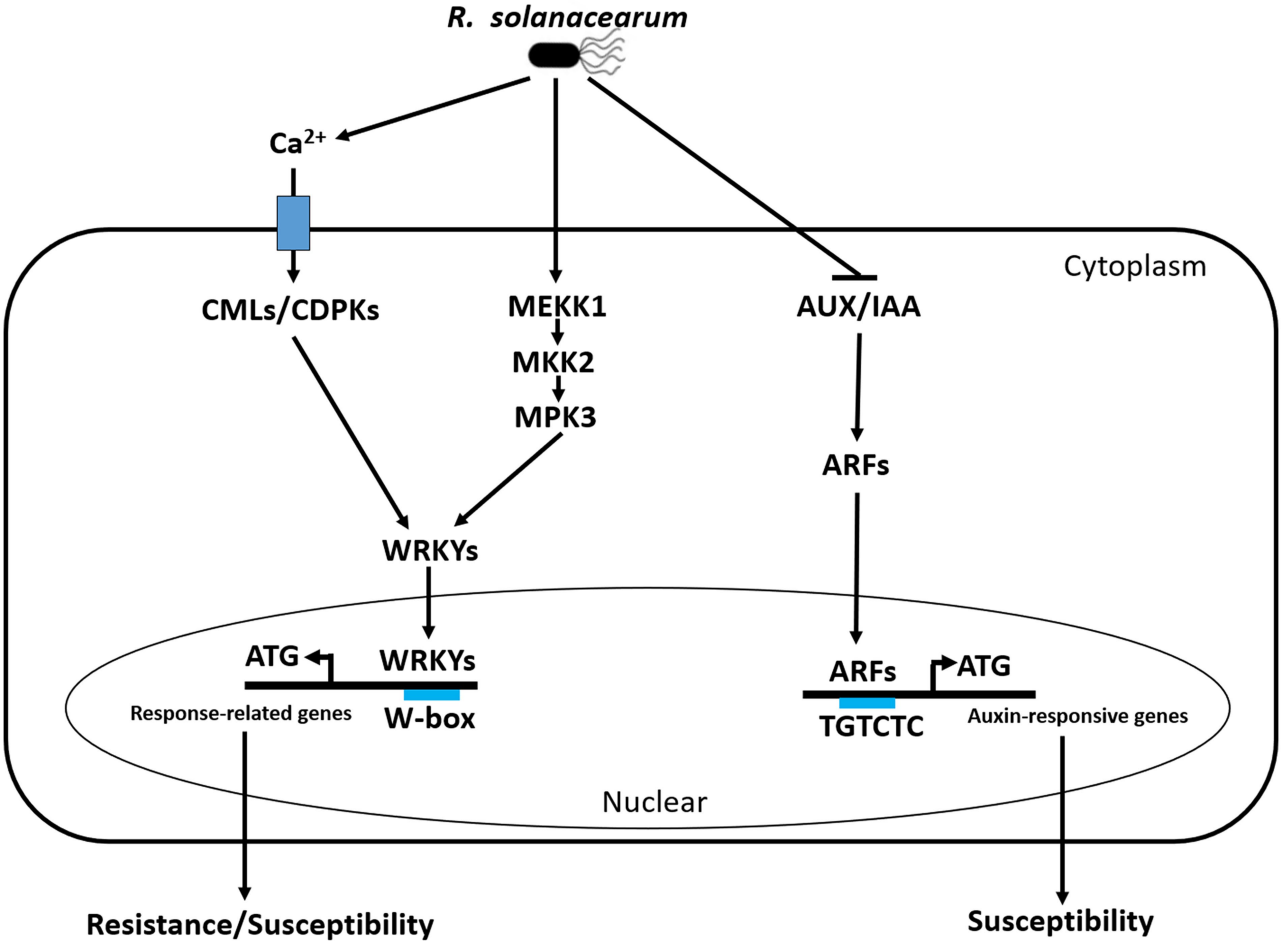
Simple schematic diagram of the peanut response to *R. solanacearum* in our study. Three early molecular events happened in the peanut response to *R. solanacearum*, including Ca^2+^ activate CMLs/CDPKs-WRKY module to regulate the expression of resistance/susceptibility-related genes, auxin signaling was induced by AUX/IAA-ARF module to activate auxin-responsive genes that contribute to susceptibility, and MEKK1-MKK2-MPK3-WRKYs was activated by phosphorylation to induce the expression of resistance/susceptibility-related genes. During the interactions between peanut and *R. solanacearum*, CMLs are possibly targeted by T3Es, CDPKs are activated by PAMPs, MAPK cascade is induced by PAMPs and T3Es, and auxin signaling is possibly activated by T3Es. CML, calmodulin-like (CML) proteins; CDPK, calcium-dependent protein kinase; WRKY, WRKY transcription factor; MEKK1, MAPK Kinase Kinases 1; MKK2, MAPK Kinases 2; MPK3, MAPK3.

Breeding of resistant cultivars is the most efficient strategy to control BW that causes serious yield losses in peanuts worldwide. A convenient and reliable evaluation method is necessary to evaluate the sensitivity of peanut to *R. solanacearum* and help to elucidate the molecular mechanism of the peanut response to this pathogen. We established and optimized a simple cutting leaf method to evaluate BW of peanut plants, which is more useful to better understand the interaction between peanuts and *R. solanacearum*. It is the first time to analyze the transcriptome changes in leaves of the resistant and susceptible peanuts infected with *R. solanacearum* by the cutting leaf method at different time points. The KEGG enrichment analysis showed that plant-pathogen interaction, plant hormone signal transduction, and MAPK signaling pathway were the outstanding pathways. Through the transcriptome profiling, we revealed that CMLs/CDPKs-WRKY module, MEKK1-MKK2-MPK3 cascade, and auxin signaling played important roles in the peanut response to *R. solanacearum*. Upon RSI, three early molecular events were possibly induced in peanuts: Ca^2+^ activate CMLs/CDPKs-WRKYs module to regulate the expression of resistance/susceptibility-related genes, auxin signaling was induced by AUX/IAA-ARFs module to activate auxin-responsive genes that contribute to susceptibility, and MEKK1-MKK2-MPK3-WRKYs was activated by phosphorylation to induce the expression of resistance/susceptibility-related genes (Figure 10). The proposed hypothetical model will advance our understanding of the molecular mechanism of the peanut response to *R. solanacearum*.

## Data availability statement

The datasets presented in this study can be found in online repositories. The names of the repository/repositories and accession number(s) can be found below: NCBI – PRJNA861998.

## Author contributions

YY designed and performed the research, analyzed the data, and wrote the article with contributions of all the authors. YY and TC performed the research and analyzed the data. XD, DY, and YW provided technical assistance to YY. XW, YZ, and XT supervised the experiments. HC, QZ, XW, and XT supervised and complemented the writing. All authors contributed to the article and approved the submitted version.

## Funding

This work was supported by the Guangdong University Scientific Research Platform and Research Project (Young Innovative Talents; grant no. 2021KQNCX033), the grants (nos. 32071737 and 32111530289) from the National Natural Science Foundation of China, the grants from the Department of Education of Guangdong Province (2020ZDZX1013), the Department of Science and Technology of Guangdong Province (2021A050530073), the Agricultural and Rural Department of Guangdong Province (KB1708008), the Basic and Applied Basic Research Fund of Guangdong Province (grant no. 2020A1515010243), and the grant (no. 202002010010) from Guangzhou Key Laboratory for Research and Development of Crop Germplasm Resources.

## Conflict of interest

The authors declare that the research was conducted in the absence of any commercial or financial relationships that could be construed as a potential conflict of interest.

## Publisher’s note

All claims expressed in this article are solely those of the authors and do not necessarily represent those of their affiliated organizations, or those of the publisher, the editors and the reviewers. Any product that may be evaluated in this article, or claim that may be made by its manufacturer, is not guaranteed or endorsed by the publisher.

## Footnotes

^1^ http://cutadapt.readthedocs.io/en/stable/

^2^ http://peanutgr.fafu.edu.cn/

^3^ https://daehwankimlab.github.io/hisat2/

^4^ http://www.bioconductor.org/packages/release/bioc/html/DESeq.html

^5^ https://www.genescloud.cn/home

## Supplementary material

Supplementary Figure S1 Workflow diagram for the RSI by the cutting leaf method in peanuts.

Supplementary Figure S2 The phenotype of A165 and A281 according to 1, 3, 4, 7 and 13 days post inoculation with HA4-1, *ΔhrpB* mutant and distilled water.

Supplementary Figure S3 The phenotype of A165 and A281 inoculated with *R. solanacearum* strain HA4-1 and *ΔhrpB* mutant by soil-drenching method.

Supplementary Table S3 All up-regulated or down-regulated DEGs in different comparison groups.

Supplementary Table S4 The results of KEGG enrichment analysis of resistance-response DEGs.

Supplementary Table S6 The results of KEGG enrichment analysis of susceptibility-response DEGs.

Supplementary Table S8 The results of KEGG enrichment analysis of PAMPs induced resistance-response DEGs.

Supplementary Table S10 The results of KEGG enrichment analysis of PAMPs induced susceptibility-response DEGs.

Supplementary Table S12 The results of KEGG enrichment analysis of T3Es induced resistance-response DEGs.

Supplementary Table S14 The results of KEGG enrichment analysis of T3Es induced susceptibility-response DEGs.
