# Supplementary figures and images for: Comparative transcriptome analysis revealed molecular mechanisms of peanut leaves responding to Ralstonia solanacearum and its type III secretion system mutant
Source: Front Microbiol. 2022 Aug 25;13:998817. doi: 10.3389/fmicb.2022.998817 (PMC9453164; doi:10.3389/fmicb.2022.998817)

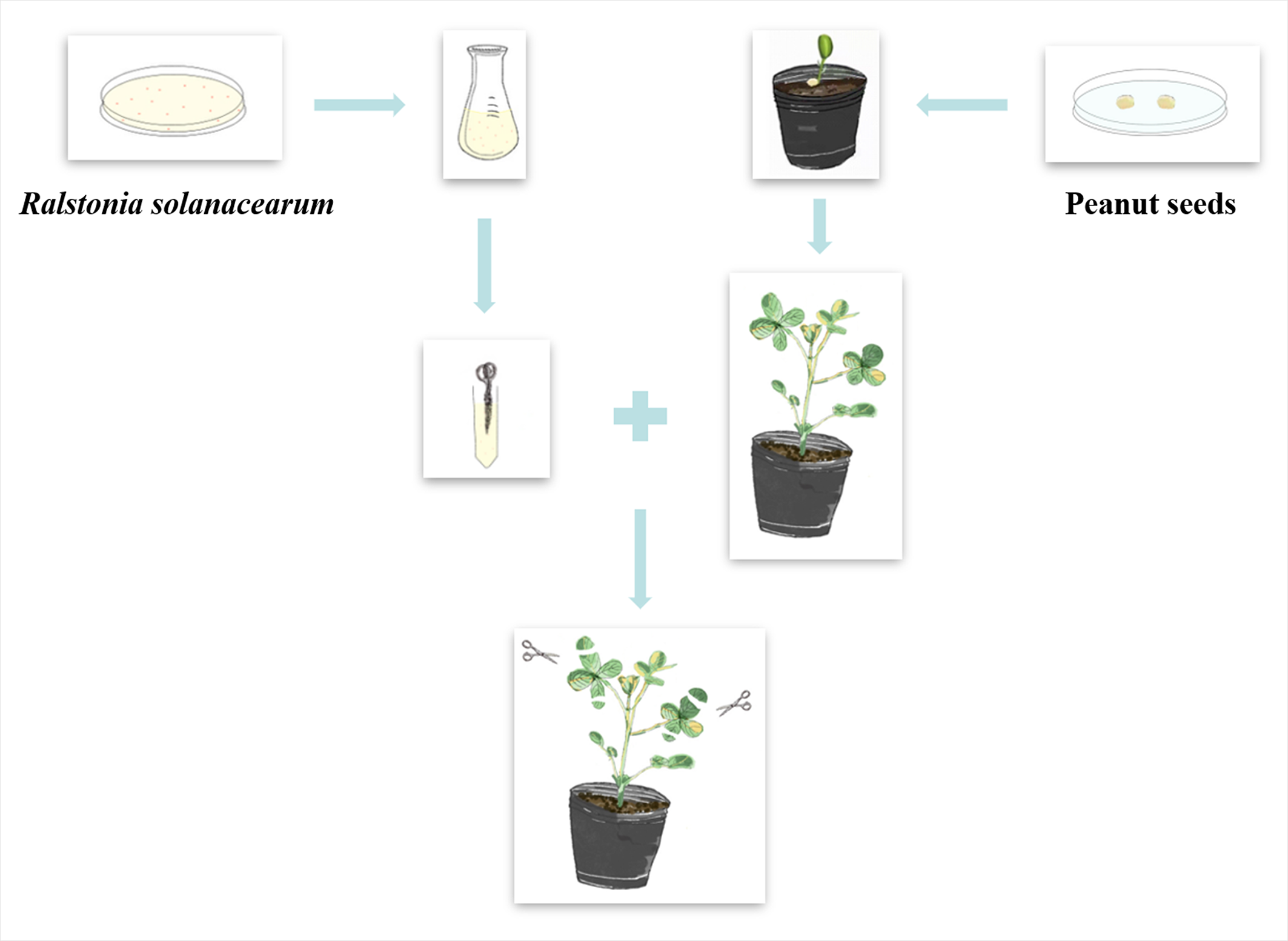

Supplement: Supplementary Figure S1 — Workflow diagram for the RSI by the cutting leaf method in peanuts. [file Data_Sheet_1.ZIP › Additional Figures/Figure S1.tif]

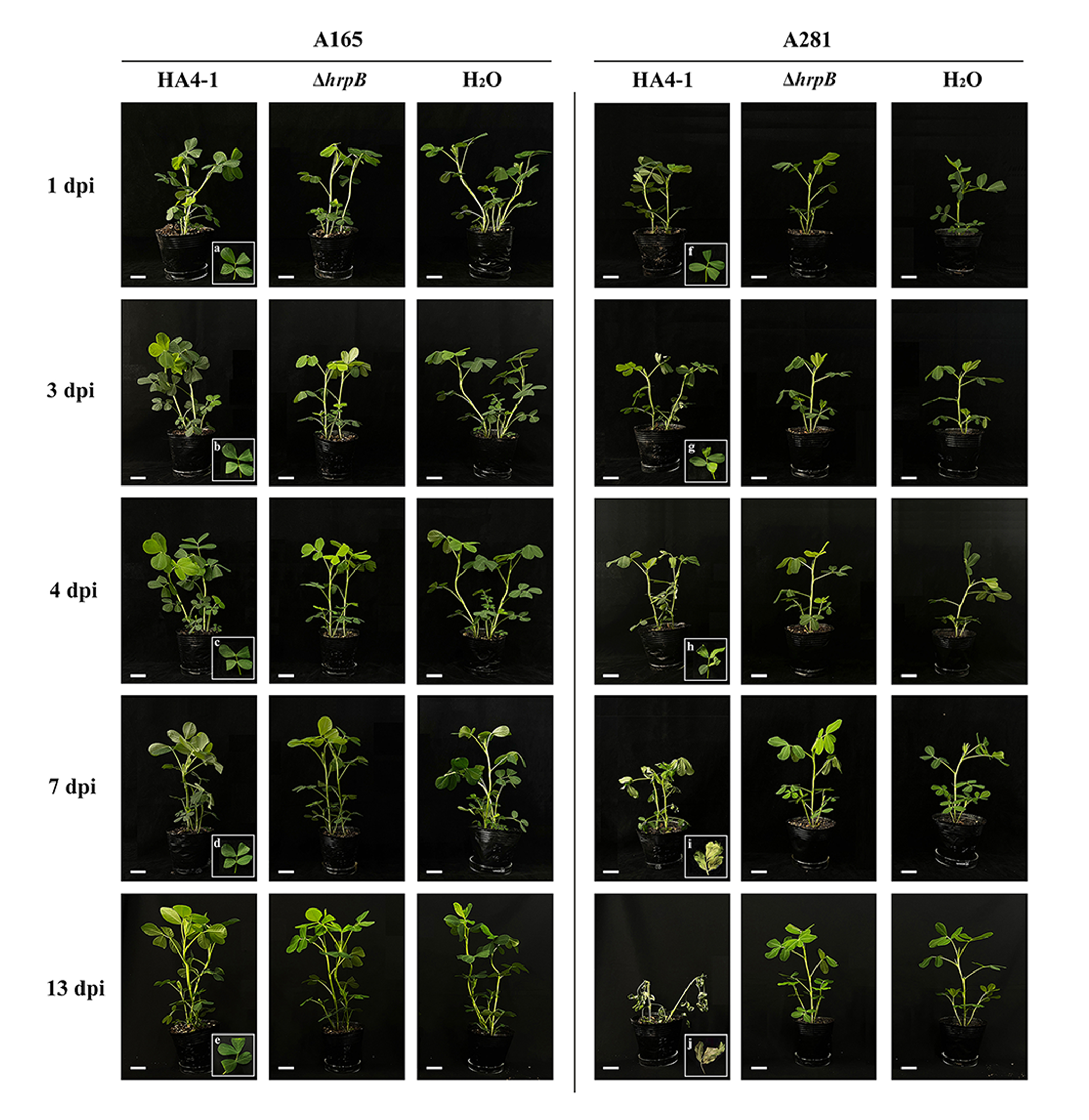

Supplement: Supplementary Figure S1 — Workflow diagram for the RSI by the cutting leaf method in peanuts. [file Data_Sheet_1.ZIP › Additional Figures/Figure S2.tif]

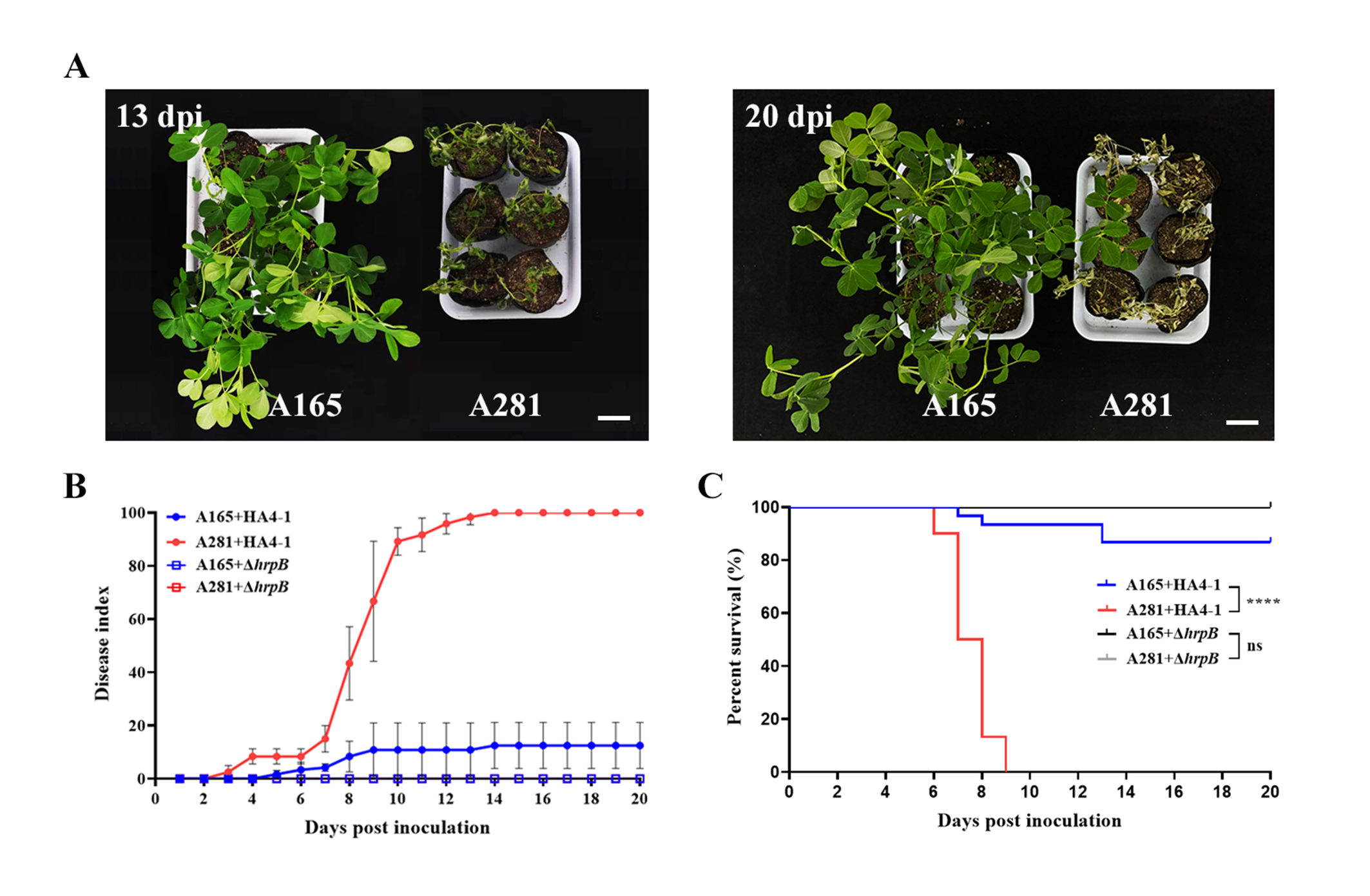

Supplement: Supplementary Figure S1 — Workflow diagram for the RSI by the cutting leaf method in peanuts. [file Data_Sheet_1.ZIP › Additional Figures/Figure S3.tif]
